# Intensive study, tuning and modification of reactive routing approach to improve flat FANET performance in data collection scenario

**DOI:** 10.1038/s41598-024-72983-y

**Published:** 2024-10-08

**Authors:** Basma M. Mohammad El-Basioni

**Affiliations:** Computers and Systems Dept, Electronics Research Institute, Elbahth Elelmy St. From Joseph Tito, El-Nozha El-Gedeeda, P.O.: 11843, Cairo, Egypt

**Keywords:** FANET, Drone, Flat routing, Reactive routing, AODV, Electrical and electronic engineering, Aerospace engineering, Computer science

## Abstract

The Flying Ad-hoc Network (FANET) can be defined as the Ad-hoc network that connects unmanned aerial vehicles flying in the space with each other and with a ground base station. However, the 3D movement of these drones with higher speeds results in a network of highly dynamic topology and intermittent connections, making the standard Ad Hoc routing protocols are not suitable for FANET. The approaches followed to address this issue include designing from scratch a routing protocol specific to FANET or modifying the existing protocols. From the view point of reliability, accuracy, and time, it is preferable to base the work on a protocol standard. But before amending the standard, tuning its performance and applying it under suitable conditions may be satisfactory for the new use. Therefore, this work considers flat FANET of fully mission-controlled drones and performs an extensive parametric simulation study to determine the best conditions and parameters’ values for applying the popular Ad Hoc On-demand Distance Vector (AODV) to it. After deducing the recommended operating environment (FAODVN-OE), some examples of amendments were suggested to further improve the performance. It was found that the modified FAODVN-OE achieves high performance compared to the default standard in terms of jitter and delay. It helped reduce jitter and delay by an average of 93.2% and 83.8%, respectively, while exhausting less energy; however the network experiences a 24.5% reduction in packet delivery ratio.

## Introduction

FANET is a type of Mobile Ad-hoc Network (MANET) and it represents a self-organizing Ad Hoc networking among unmanned aerial vehicles known as drones may be equipped with different sensors, cameras, actuators, and GPS, connected to ground station(s) or other types of networks allowing for real-time data collection and control over inaccessible areas. With these characteristics and capabilities, FANET can formulate endless application scenarios in different application fields, but at the same time this imposes challenges on operating and maintaining such network.

These challenges are related to the restrictions on drones hardware capabilities, the nature of drones communication medium, mission requirements, and the drones operating environment.

One of the biggest challenges that faces the realization of FANET systems is achieving long-endurance missions with battery-operated drones. Comes after it the challenge of mobility management and trajectory optimization. Another important challenge is the obstacle detection and collision avoidance. In addition to that finding accurate position information of each drone in harsh and indoor environments is a crucial design challenge of FANET systems. Realizing a fully autonomous flights in the absence of a fixed infrastructure is a big challenge. Achieving the required quality of service and quality of experience is also a big challenge. 3D movement of drones imposes great complexity in FANET design. Last, but not least, the security of FANET transactions is very important; achieving confidentiality, authentication, authorization, non-repudiation, and data integrity in such highly dynamic nature is a nontrivial task. FANET inherits all the security attack types from Ad-hoc Networks such as, eavesdropping, spoofing, false routing message, rushing, and gray hole, while the highly dynamic low-density nature of FANET makes it is more difficult to correctly identify these attacks and attackers.

In order to overcome all the above mentioned challenges, special consideration should be paid to hardware design of drones such as using directional antenna instead of omni-directional antenna, flight planning such as optimal tasks assignment, and developing energy-efficient and non-complex protocols suitable for drone’s low processing and storage capabilities. We may see these challenges especially in the absence of networking standards specific to FANET.

The design of FANET includes many aspects, including: path planning^1,2^, topology control^3,4^, medium access control protocol^5,6^, routing protocol^7,8^, mobility models^9^, joint design between protocols^10,11^, etc.

The highly dynamic nature of FANET entails performing adjustments of MANET standards to be suitable for this nature. Perhaps the thing most affected by this is the design of the routing protocol because of the inherent instability of routes. The highly dynamic network topology of FANET requires more flexibility in constructing data routes between sources and destinations, in addition to that most of the time its application features real-time communication requires low overhead and low latency; this makes the flat network structure and reactive routing are more logical for FANET implementation. The most popular reactive routing protocol, AODV, was selected to represent reactive routing in this work.

AODV is characterized by reduced overhead, low energy consumption, support of different mobility rates, and better network scalability^12^. However, it suffers from delay, and in small data rates, the routes may be expunged from the table even if no change in topology was occurred. Moreover, frequent link failure in FANET leads to increased route discovery times and thus increased overhead and generally degrades the network performance^13^.

The design of a standard routing protocol includes defining message types, specifying message formats, operation and procedures, timing constraints and sequence charts, parameters and their ranges and default values, etc. Amending the standard can be through modifying any or a combination of these design determinants.

In our opinion, the most influential thing on the routing performance is choosing the values of its parameters, which is the first thing that can be done to help adjust performance to the needs of the usage scenario, and may eliminate the need for making modifications in its operation or at least provide the basis for any further modification. Based on this vision, the present work incorporates a parametric study of AODV for FANET.

The network behavior was tested against different operating conditions: drone density, speed, and transmission interval, to determine the best conditions of applying AODV to FANET. Then, parametric analysis was conducted on key parameters and the parameters that controls main AODV operations. The effect of applying the standard HELLO mechanism was also analyzed, then a recommended Operational Environment (OE) of AODV-based FANET (FAODVN) was deduced. The contributions of this paper include:

- Charting the flow of operations of AODV process to be more understandable helping in its implementation and modification.
- Using a common scenario of FANET applications, a simulation study was conducted to find the preferred conditions for applying AODV to FANET with respect to drones density, drones speed, and drones transmission interval.
- In the study, the behavior of the network wasn’t only be tested in terms of the main performance metrics such as average end-to-end delay, data loss, and energy consumption, but the primary parameters that control the behavior of these metrics and thus indirectly affect the performance of the network were considered for deeper analysis of the network behavior, needs, and possible enhancements.
- Parametric study was conducted for tuning the network performance. This study includes the key independent parameters that control the value of other important parameters, and the parameters related to the main operations of the protocol such as RREQ dissemination control.
- Testing the viability of using the optional HELLO mechanism after tuning the network.
- Proposing modifications to the tuned AODV to further improve the performance.
- The recommended FAODVN-OE together with the proposed modifications were proved to give better performance over using the standard default values in terms of delay, jitter, and energy consumption.

The rest of this paper is organized as follows; Section "Background", gives schematic explanation of AODV protocol as generic or basic reactive routing protocol; Section "Previous Work", reviews previous work; Section "Methodology", illustrates the research methodology; Section "Performance Assessment Experiments", describes the details of the conducted assessment experiments; Section "Results and Discussion", presents and discusses the obtained results; finally, Section "Conclusions and Future Work", concludes the paper and shows the direction of future work.

## Background

**Fig. 1:**
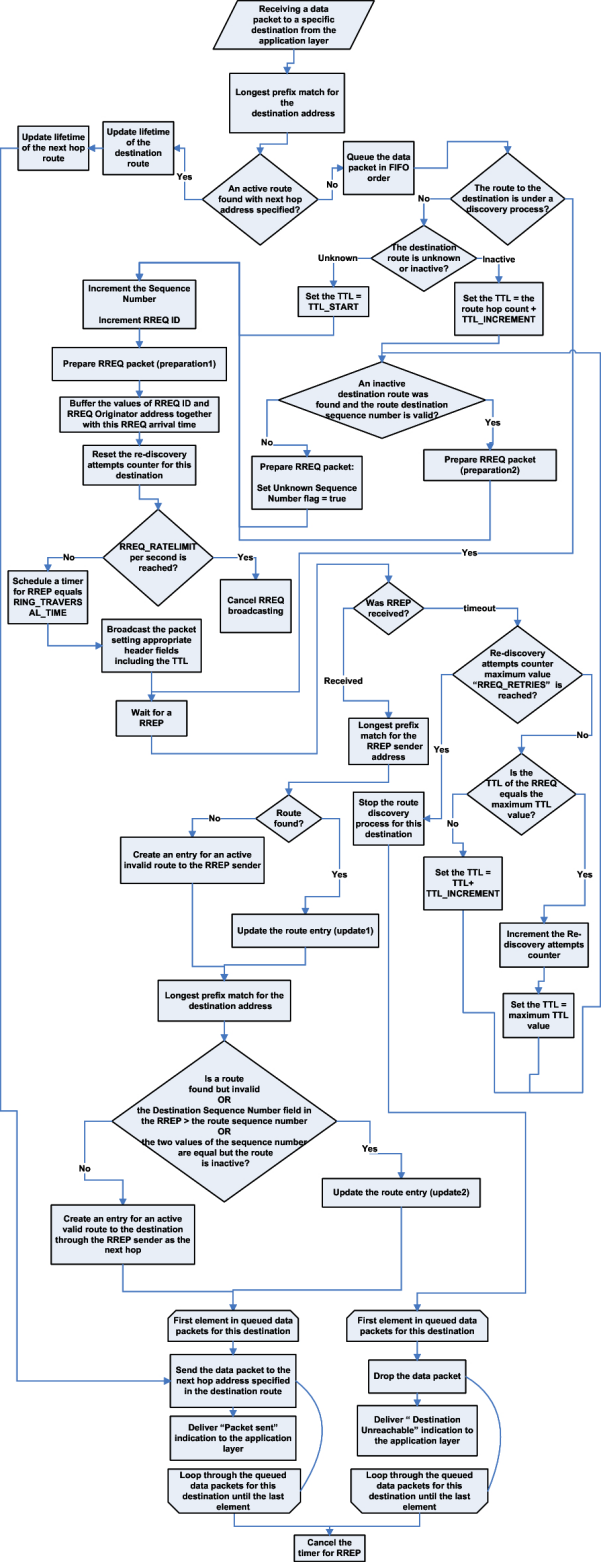
Operations of AODV route discovery originator node. This flowchart visually illustrates the details of the operations performed by a node wants to transmit a message and has no route to the intended destination, therefore it performs an AODV route discovery. It assumes a knowledge of AODV basics and standard specifications.

**Fig. 2:**
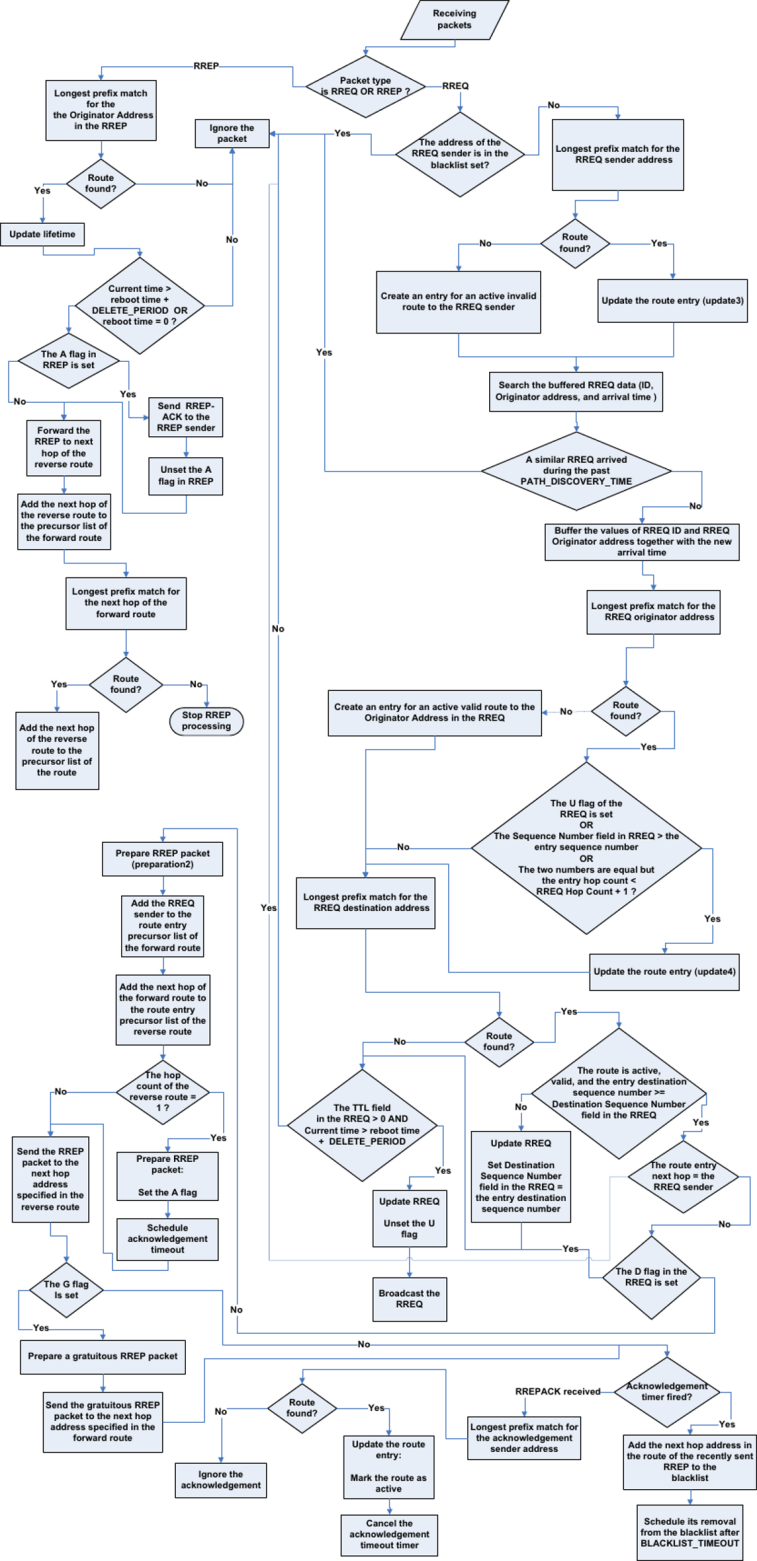
Operations of an AODV intermediate node. This flowchart visually illustrates the details of the operations performed by a node that receives the RREQ message and is not the destination of the AODV route discovery process. The flowchart assumes a knowledge of AODV basics and standard specifications.

**Fig. 3:**
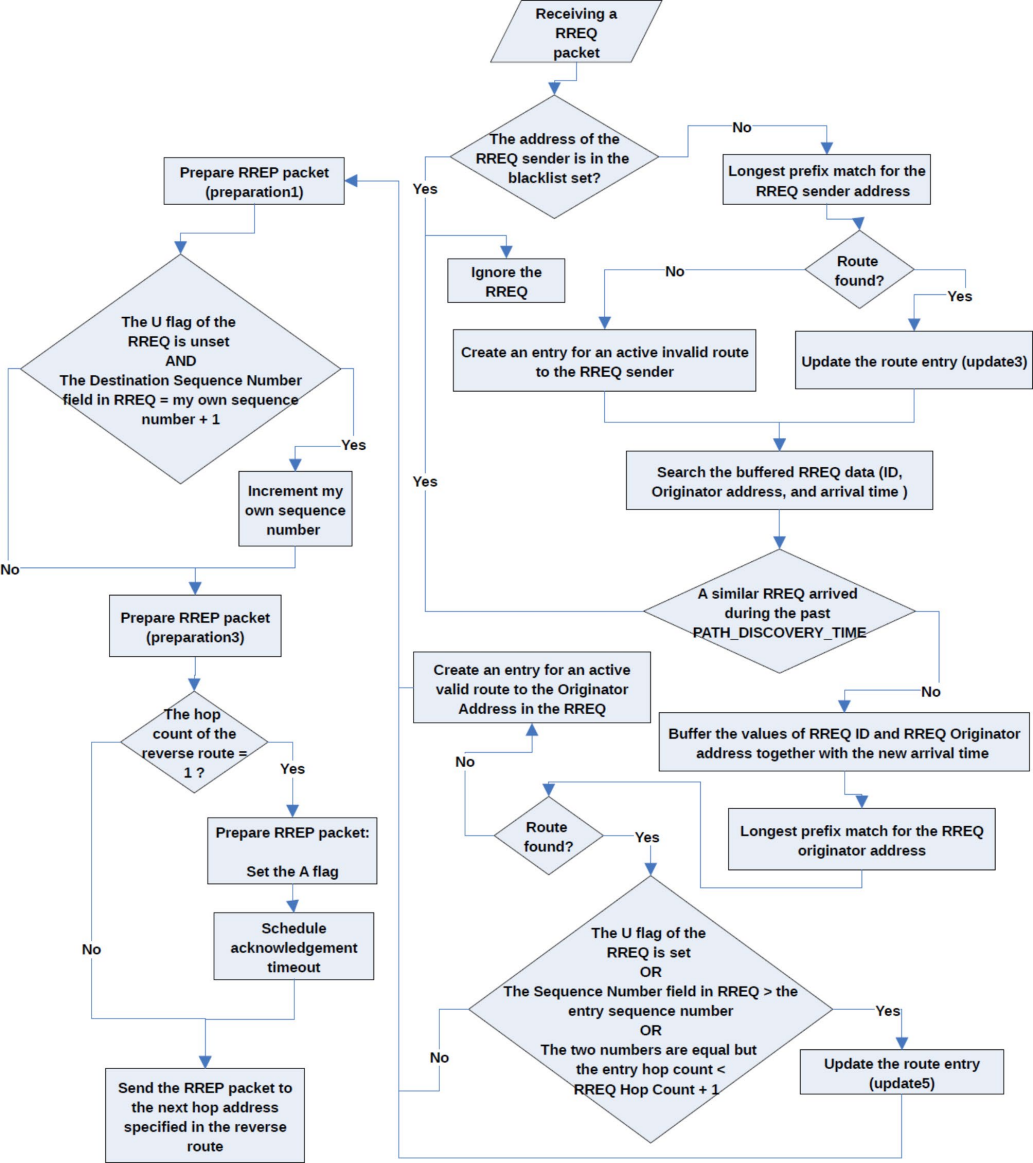
Operation of a destination node in AODV. This flowchart visually illustrates the details of the operations performed by a node receives a RREQ message and it was the destination of the AODV route discovery process. The flowchart assumes a knowledge of AODV basics and standard specifications.

**Fig. 4:**
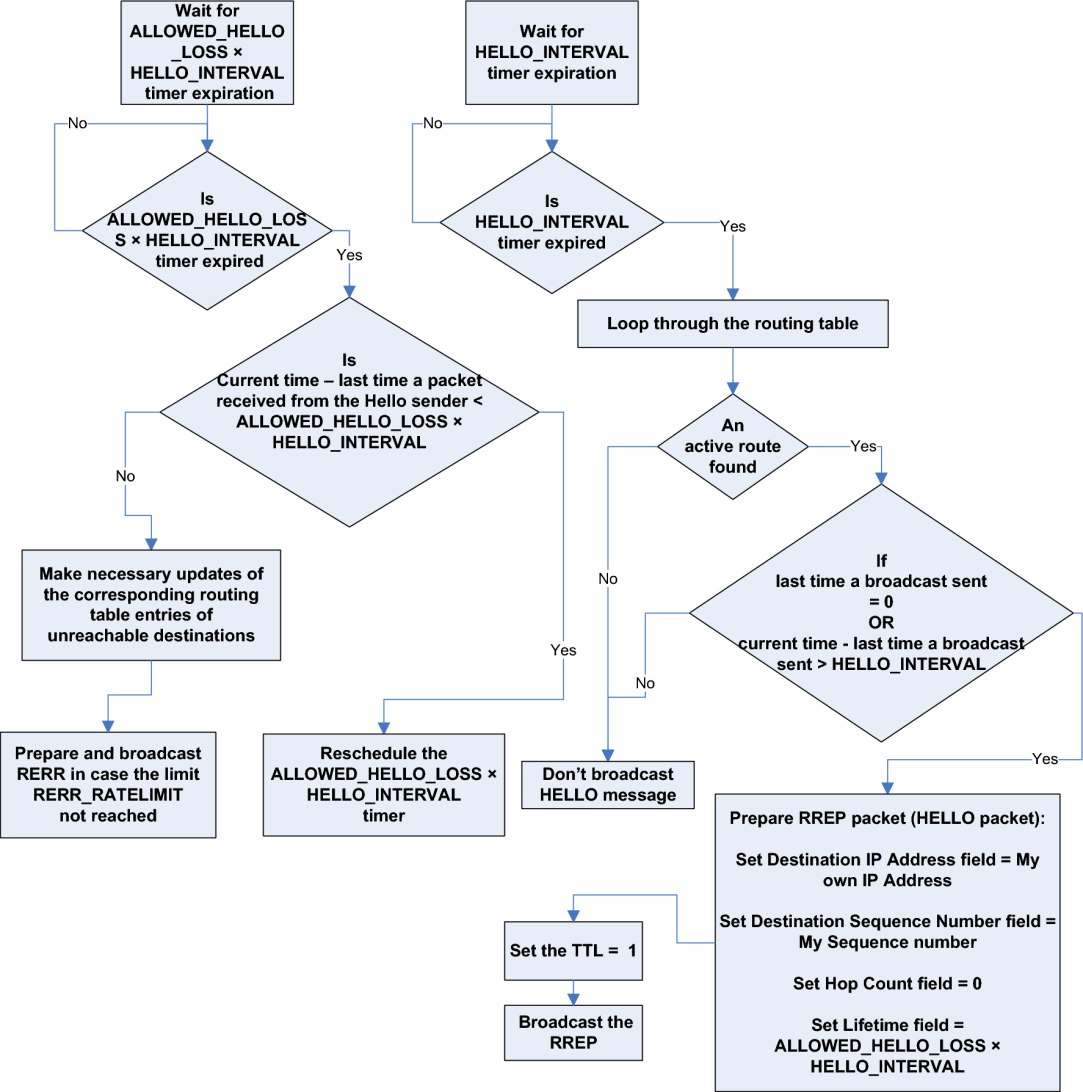
The HELLO mechanism procedures in AODV. This flowchart visually illustrates the details of the HELLO-related procedures performed by a node, which periodically broadcasts Hello messages, to keep track of link state of neighbor nodes. The flowchart assumes a knowledge of AODV basics and standard specifications.

This section is intended to give a background on AODV as generic or basic reactive routing. It assumes a knowledge of AODV basics and standard specifications, and it was enough to visually illustrates the details of its operations. Therefore, the flowcharts undefined Figs. 1,  2, 3, 4 connect, focus and present the main operations of AODV in terms of the role of the network node. Figure 1 illustrates the operations of route discovery originator node, Fig. 2 illustrates the operations of an intermediate node, Fig. 3 illustrates operation of a destination node, while the illustration of the HELLO mechanism procedures is separated in Fig. 4. To make the figures simpler and more comprehensible, some terms were used in them and their definitions are as follows:

Longest prefix match: perform longest prefix match for an address in the routing table to find a corresponding route.

Update lifetime: update a route lifetime in the routing table to MAX( current lifetime, current time + ACTIVE_ROUTE_TIMEOUT).

Update the route entry (update1): Set the entry route lifetime = current time + ACTIVE_ROUTE_TIMEOUT, Set the entry hop count = 1.

Update the route entry (update2): Set the next hop = RREP sender, Set the entry hop count = Hop Count field in the RREP + 1, Set the entry destination sequence number = Destination Sequence Number field in the RREP, Set the entry route lifetime = current time + Lifetime field in the RREP, Mark the route as active, Mark the route as valid.

Update the route entry (update3): Set the entry destination sequence number = Originator Sequence Number field in the RREQ, Set the entry route lifetime = current time + ACTIVE_ROUTE_TIMEOUT, Set the entry hop count = 1, Mark the route as active, Mark the route as invalid.

Update the route entry (update4): Set the entry next hop = RREQ sender address, Set the entry destination sequence number = MAX(Sequence Number field, entry sequence number), Set the entry route lifetime = MAX(current time, MinimalLifetime), Set the entry hop count = RREQ Hop Count + 1, Mark the route as active, Mark the route as valid.

Update the route entry (update5): Set the entry destination sequence number = MAX( Sequence Number field, entry sequence number), Set the entry route lifetime = MAX(current time, MinimalLifetime), Set the entry hop count = RREQ Hop Count + 1, Mark the route as active, Mark the route as valid.

Create an entry for an active invalid route to the RREP sender setting: the entry destination sequence number = Destination Sequence Number field in the RREP, the entry route lifetime = current time + ACTIVE_ROUTE_TIMEOUT, the entry hop count = 1.

Create an entry for an active valid route to the destination through the RREP sender as the next hop setting: the entry hop count = Hop Count field in the RREP + 1, the entry destination sequence number = Destination Sequence Number field in the RREP, the entry route lifetime = current time + Lifetime field in the RREP.

Create an entry for an active invalid route to the RREQ sender setting: the entry destination sequence number = Originator Sequence Number field in the RREQ, the entry route lifetime = current time + ACTIVE_ROUTE_TIMEOUT, the entry hop count = 1.

Create an entry for an active valid route to the Originator Address in the RREQ setting: the entry next hop = RREQ sender address, the entry destination sequence number = Originator Sequence Number field in the RREQ, the entry route lifetime = MAX(current time, MinimalLifetime), the entry hop count = Hop Count field in the RREQ + 1.

Prepare RREQ packet (preparation1): Set Originator IP Address = local IP address, Set Originator Sequence Number field = Sequence Number, Set Destination IP Address field = this destination IP address, Set Hop Count field = 0, Set RREQ ID field = RREQ ID, Set other flags appropriately.

Prepare RREQ packet (preparation2): Set Destination Sequence Number field = route destination sequence number, Set Unknown Sequence Number flag = false.

Prepare a gratuitous RREP packet: Set Destination IP Address field = Originator IP Address field in the RREQ, Set Originator IP Address field = Destination IP Address field in the RREQ, Set Destination Sequence Number field = reverse route sequence number, Set Lifetime field = reverse route lifetime, Set Hop Count field = reverse route hop count.

Prepare RREP packet (preparation1): Set Destination IP Address field = Destination IP Address field in the RREQ, Set Originator IP Address field = Originator IP Address field in the RREQ.

Prepare RREP packet (preparation2): Set Destination IP Address field = Destination IP Address field in the RREQ, Set Originator IP Address field = Originator IP Address field in the RREQ, Set Destination Sequence Number field = forward route sequence number, Set Hop Count field = forward route hop count, Set Lifetime field = forward route lifetime – current time.

Prepare RREP packet (preparation3): Set Destination Sequence Number field = my own sequence number, Set Hop Count field = 0, Set Lifetime field = MY_ROUTE_TIMEOUT.

## Previous work

Several research in the previous literature have dealt with the application of Ad Hoc reactive routing protocols to FANET. Some research articles, such as^12,14^, have focused on doing performance comparisons among AODV and other proactive routing protocols in the context of FANET. They performed simulation experiments to assess the network performance evaluation metrics: packet delivery rate, end-to-end delay, throughput, and energy consumption with varying some parameters such as drones’ speed and antenna height; but they have some shortcomings such as using a rather small and constant number of drones for the evaluation. They ignored considering the effect of different mobility and traffic patterns. The performed comparisons can be described as simple and incomprehensive, didn’t give justifications, illustrations, and significant performance suggestions.

The performance comparisons, especially that are based on small networks, tend only to give the logical meaning and the expected results for the comparison based on the known characteristics of AODV, for example, it is expected that the reactive AODV protocol is better in terms of metrics such as packet delivery rate and energy consumption, and gives low performance than proactive protocols with respect to delay.

It is worth noting that some other few performance comparisons such as the work in^15^ give a deeper comparison with explanations and conclusions, where it investigated the ability of AODV to find and maintain the routes in the relaying FANET using relatively larger number of drones, but also constant throughout the simulation runs. It evaluated other performance measures such as routing overhead metrics, average hop count, jitter on varying different parameters such as speed and packet size.

In general, it could be said that most of the comparison work concluded the better performance of the reactive AODV against using proactive approaches except in terms of jitter, end to end delay and hop count metrics, and especially for highly dynamic and low density networks in open air FANET scenario. However, it could also be said that the performance comparison research articles didn’t give a solid base for future development regarding the application of AODV in FANET.

Few other relatively old research articles have focused on performing experimental analysis to test the functionality of AODV in FANET, may be for specific application scenario and for improving certain metric. For example, the work in^16^ studied the dependence of drones connectivity on the AODV network parameters—drones number, speed, and transmission range—and how these parameter values can be determined in order to achieve a certain high percentage of packet delivery ratio taking into account the effect of different drone speeds. From the simulation results, the paper concluded optimal values of the network parameters for the chosen network topology, data transmission technology, and traffic parameters for achieving the required performance goal.

The result is specific to the employed scenario setup, and it would have been better, for the sake of more reliable results, if the experiments setup had been more precise and comprehensive testing larger ranges of parameters’ values with more granular resolution. The paper also studied packets dropping to determine the dominant factors out of several factors causes packet loss in the simulation.

A lot of old and recent works have proposed routing protocols for FANET that represent modification on AODV. The work in^17^ modified the path selection metric to consider an expected connection time and node failure probability in addition to the hop count. New information was contained in AODV control packets: the position, velocity, maximum risk value, and minimum expected connection time. The drone calculates the expected connection time and the risk value based on the sender’s and its own position and velocity values. The routing table is updated with every new information received. It employed a hybrid directional and omnidirectional routing technique with dynamic angle adjustment mechanism and a dependant local path repair mechanism.

The work in^18^ proposed also a new metric for the estimation of link stability from the mobility prediction represented by the next position of the drone, safety degree in terms of the closeness of the drones, and link quality information. These values were computed based on the position information added to the periodical Hello packet. Additionally, this work used proactive route maintenance strategy to adapt to the instability of paths due to the highly dynamic nature of FANET where the drone selects the most stable path but saves lower stability paths, and periodically assess the quality of the currently used path to switch paths before the current path disconnection.

The link stability also was determined in literature based on the flying directions of the two communicating drones. If they are determined to move in reverse directions, the link is assessed to be unreliable. This assessment required modifications in AODV RREQ packet to include the sender’s current position, current direction, velocity, and timestamp; and it was performed by comparing the initial and final distances between the two drones when the sender sent the RREQ and at the time of RREQ reception by the second drone, respectively.

Some work integrated AODV with geographic forwarding. The drone uses it in case of route failure or in case of receiving a data packet it has no route for it. This also needs redefinition of AODV packets to include the drones position information.

Some works^19^ dispensed with the route maintenance mechanism by route preservation mechanism uses bio-inspired mobility control techniques.

The work in^20^ addressed the issue that AODV doesn’t support path accumulation and multi-path routing. It exploited ANT Colony Optimization to modify AODV to support these techniques, and proved by simulation performance improvements in terms of packet delivery ratio and throughput, average delay, packet loss ratio, and control overhead percentage.

The cross-layer design concept was also used to improve AODV performance for FANET such as the work in^21^ that proposed cross-layer design approach between the network layer in the form of building a cluster-based topology with an energy-efficient cluster head selection and a dependent data link layer with a MAC protocol combines CSMA/CA, TDMA, and prioritization scheme, cooperatively designed to reduce links breakage.

Some recent researches have utilized machine learning techniques to improve FANET routing. The work in^22^ tackled the modification of AODV to be suitable to FANET such that it results in more stable paths and higher packet delivery rate. It was also concerned with achieving load-balance among drones and maximizing the network lifetime, thus it introduced new parameters for the route selection process comprises route delay and residual energy information besides the common information of link quality, motion direction, and distance. It was composed of two phases: a Q-learning-based route discovery process, and a route maintenance process in which the drone tests its energy and traffic levels against specified thresholds, and tests its connections quality to detect a problematic path that should be altered.

To the best of our knowledge, no paper studied tuning the parameters of the commonly used AODV protocol for FANET scenario with a good degree of comprehensiveness instead of first resorting to making modifications to its operations. It seems useful to introduce a routing protocol suitable for FANET doesn’t require complex implementation, implemented as possible as it could be from merely adjusting a standard protocol parameters, ensuring compatibility with other devices and systems; hence, this paper tries to address this issue with a deeper analysis and in a more comprehensive manner.

## Methodology

The work in this paper supports using the standard protocols of Ad Hoc networks for FANET in contrast to the tendency to design from scratch non-standard protocols specific to FANET to produce FANET-specific standards. This may be done by adjusting the standard by selecting between its design options, tuning its parameters’ values, setting its reserved flags, formatting messages’ payload, etc. Subsequently, if a need exists, this can be accompanied by simple amendment to the standard to further improve the performance. The proposed work is also based on the fact that the protocol may not be suitable to all network conditions, or in other words, a specific network condition gives the protocol best performance, especially with great diversity of network conditions.

From this standpoint, this work aims to study the application of AODV routing protocol in FANET. The effect was evaluated using the main network performance metrics, and other metrics were used for deep analysis and understanding. To have flexibility in formulating the scenario of FANET analysis, employing variety of operating conditions, and have full control over network and protocol parameters for deeper analysis, the discrete event simulation is used to produce and analyze the data. Also, simulating the network is an important step before the real deployment of the network design, especially when the network entities are drones, to avoid problems and damages, save time, and facilitate testing with real deployments, besides improving the performance.

**Fig. 5:**
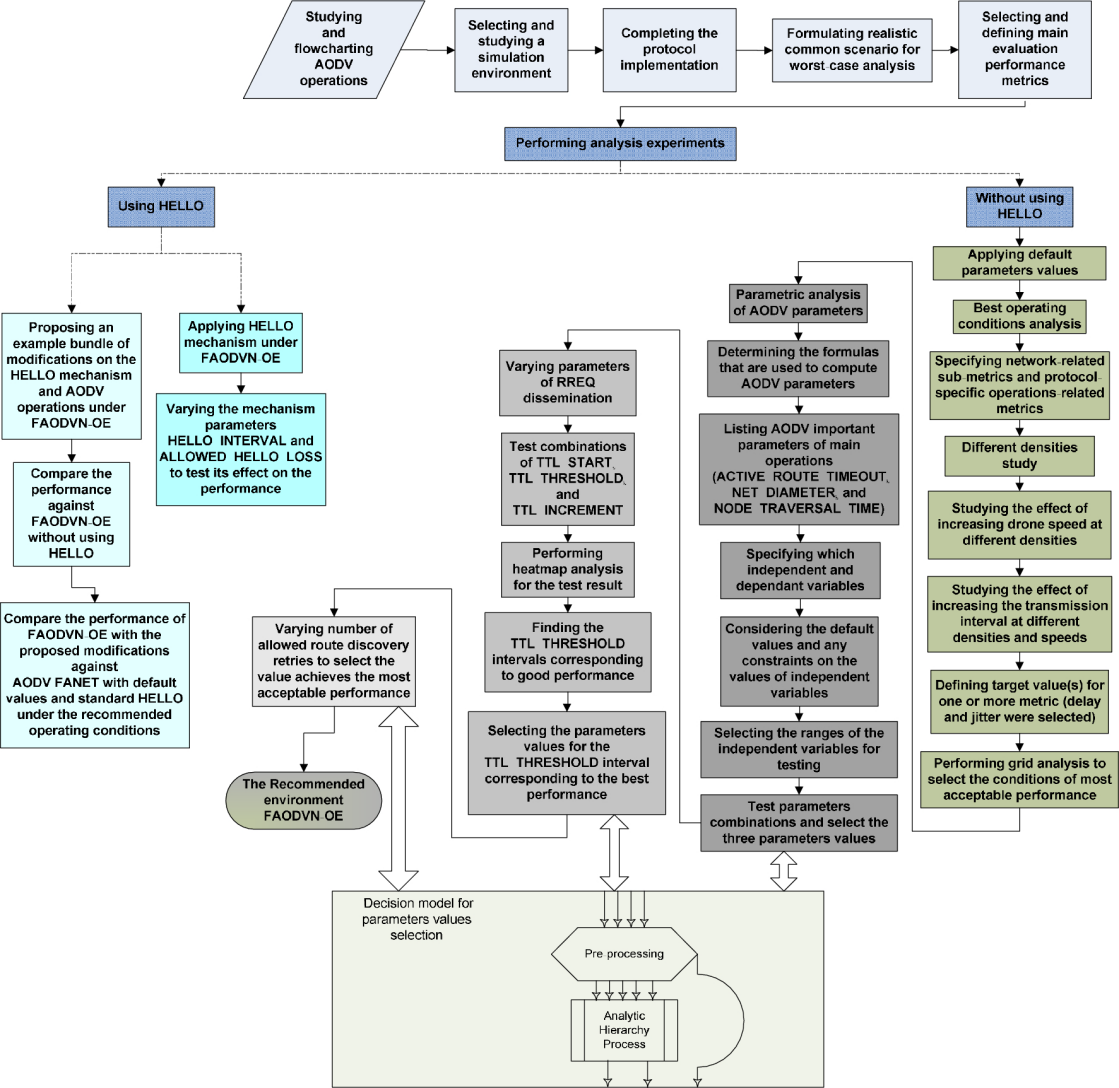
An illustration summarizing the research methodology in this work. This diagram visually illustrates the rationale and sequence of phase and steps taken to produce the work proposed in this paper. The first stage is the preparation stage where it incorporates the studying the details of AODV protocol, along with selecting and setting up the environment of the simulation analysis. The performance analysis stage includes two main experiments: analysis employing HELLO mechanism and analysis without using it. The analysis without using HELLO is performed first, and it has sub-stages represent finding the best operating conditions, then performing parametric analysis of AODV parameters until finding the recommended operational environment FAODVN-OE for using AODV in FANET. The effect of the HELLO mechanism on AODV performance, under the recommended operational environment, is analyzed in two different forms: the standard form and a modified version. Each stage and sub-stage in the diagram is highlighted in different colors.

The followed research methodology: the simulation experiments setup basis, selecting independent and dependent variables, order of analysis stages and steps, etc. can be summarized with the diagram in Fig. 5.

Of course the preparation stage of the experimental analysis started by studying the standard and using flowcharting method for accurate understanding and effective analysis and documentation. The preparation stage contains also selecting a famous reliable discrete event simulator and a simulation framework for AODV implementation. This framework and its AODV implementation were studied well, tested, and completed, such as completing the implementation of the HELLO mechanism. The last step in the preparation stage is putting the network model and assumptions for worst-case analysis of a realistic common FANET scenario or part of a common scenario, and selecting and defining main evaluation performance metrics to reflect: the reliability of the network, quality of service and user experience, and the effect of the achieved performance on the drones energy and accordingly the mission duration.

In the beginning, the experiments were done without using the HELLO mechanism. The early study aims to find the best operating conditions, so that the default parameter’s values were applied. Network-related sub-metrics, such as hop count and path failure times, as well as protocol-specific operations-related metrics, such as number of RREQ sent, path discovery failure times, and route finding time, were evaluated for accurate analysis and to give insights and suggestions about the protocol design. Cisco recommended values of delay and jitter for real-time applications were used to determine target values of the two metrics.

The combinations of different operating conditions were tested at three stages: in the first stage, the range of drone speed and transmission interval were kept constant while the drone density varied; in the second stage, the speed range varied at constant transmission interval range and at different values of drone density selected from the result of the first stage; and in the third stage, the transmission interval range became the varying condition at certain values of speed and density selected based on the observation of the second stage output.

Utilizing the results of testing different combinations of density, speed, and transmission interval values, and referring to the specified target metrics values, grid analysis is used to select the conditions of most acceptable AODV-based FANET (for simplicity, it is also referred to as AODV FANET throughout the paper) performance, the conditions that should be avoided, and the others which can be used to tune performance at runtime.

The study then focused on parametric analysis by testing different combinations of parameters’ values under the best operating conditions until FAODVN-OE was deduced.

The parametric analysis is composed of different sequential steps. First, the protocol parameters were studied, the formulas used to compute them were determined, then they are categorized into parameters related to the main operation of route discovery and parameters related to non-core processes complementary to the main operation, and into dependant and independent parameters. Three independent key parameters of the protocol’s main operation, of which many other parameters are functions, were varied.

Then, the parameters used in a complementary yet important operation, which is the expanding ring search process for RREQ dissemination, were considered and their combinations were analyzed using heatmap analysis. The last tested parameter is the RREQ_RETRIES which controls the decision of reattempting discovery or ceasing discovery and dropping all packets destined for this unreachable destination.

**Fig. 6:**
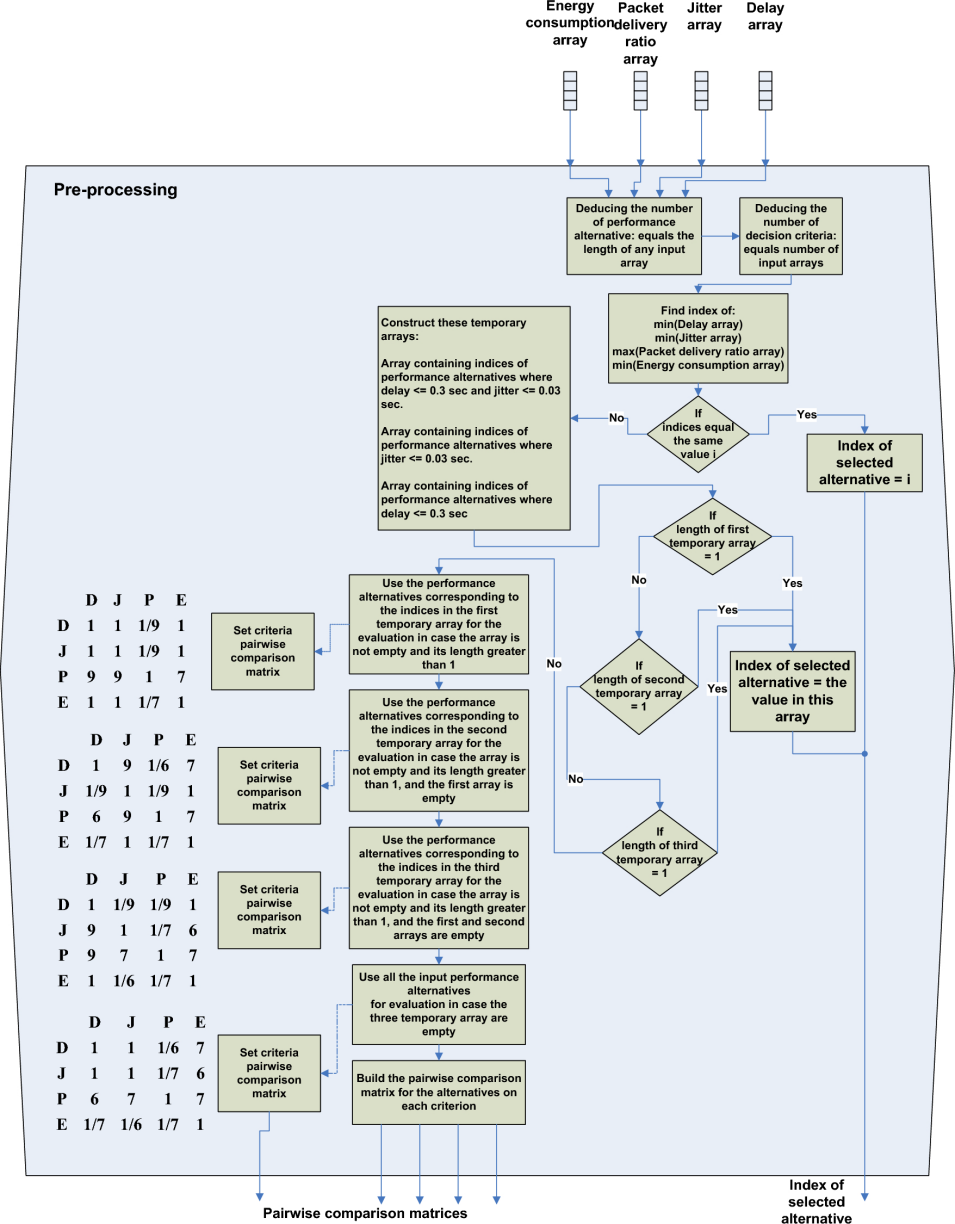
Represents the pre-processing stage of the analytical decision model proposed for parametric analysis.

In addition to visual analysis, a decision model to select parameters values is employed in the parametric analysis and composed of two main stages: the pre-processing stage and the Analytic Hierarchy Process (AHP) stage. The decision model was implemented in MATLAB. The pre-processing is the first stage of data processing and may be the last stage if the decision to choose parameters values is taken during it. As shown in Fig. 6, it takes the arrays of the four performance metrics values corresponding to different parameters combinations, and filters them upon some criteria to produce a new array for the next processing stage. It guarantees that the values considered are only the values corresponding to the combinations in which both delay and jitter conditions are met or in which one of the conditions is met. If none of these are met, it passes the entire input arrays to the next stage.

In general, the pre-processing takes the final decision to choose a specific array index for a parameters combination as the combination that achieves the required performance if it finds that this combination achieves the lowest delay, jitter, and energy consumption values and the largest PDR value, or it is the only one that fulfills one or both of the delay and jitter conditions.

If data processing proceeds to the AHP stage, the pre-processing prepares the pairwise comparison matrices; it sets the criteria pairwise comparison matrix according to the type of the filtered data. For example, in case the filtered data elements meet the two conditions, delay and jitter as well as energy consumption criteria are set to be equally preferred, while the PDR is given the most importance. since the delay and jitter limits are already satisfied, the PDR criterion is set to be extremely preferred than them and very strongly more important than the energy consumption criterion.

**Fig. 7:**
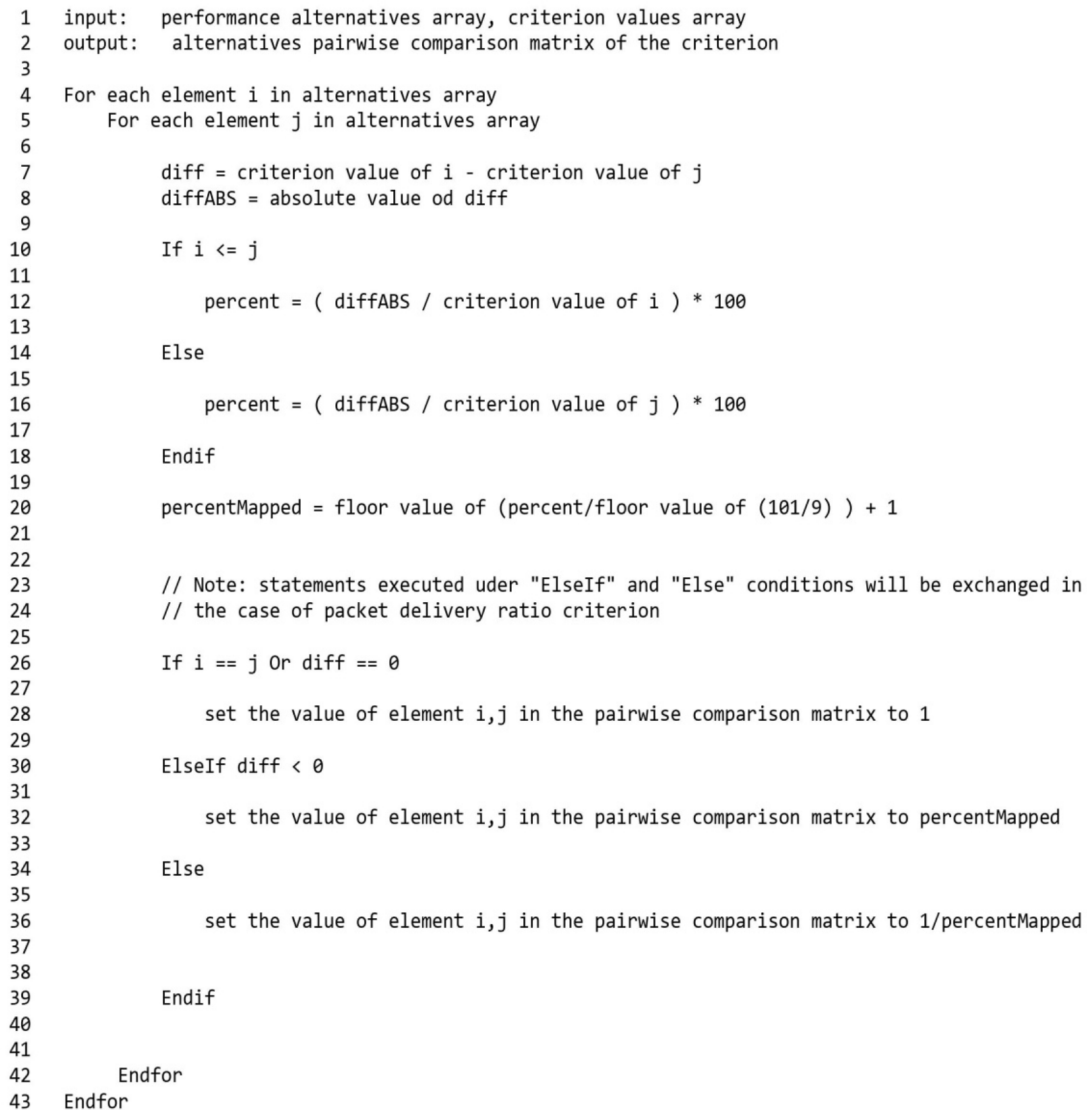
Is the process of building a pairwise comparison matrix on a specific criterion for alternative combinations of performance.

The pairwise comparison matrix for the alternative combinations on each criterion is subsequently built by the pre-processing stage from the criterion values after filtering. Figure 7 illustrates this process for a specific criterion where the inputs represent the performance alternatives array, which is an array contains the indices of the filtered elements, and the input array of the criterion values. It is important to mention that each index represents a parameters combination and it is used for lookup, where it can be referenced to match criteria against the parameters combination producing it and thereby the values of a specific parameters combination and its corresponding values from the input criteria arrays can be retrieved.

The relative importance of one performance alternative over another alternative on a criterion is calculated in terms of the percentage increase or reduction in the value of the criterion that it produces versus the criterion value produced by the other one, with this percentage set on a nine-point scale.

**Fig. 8:**
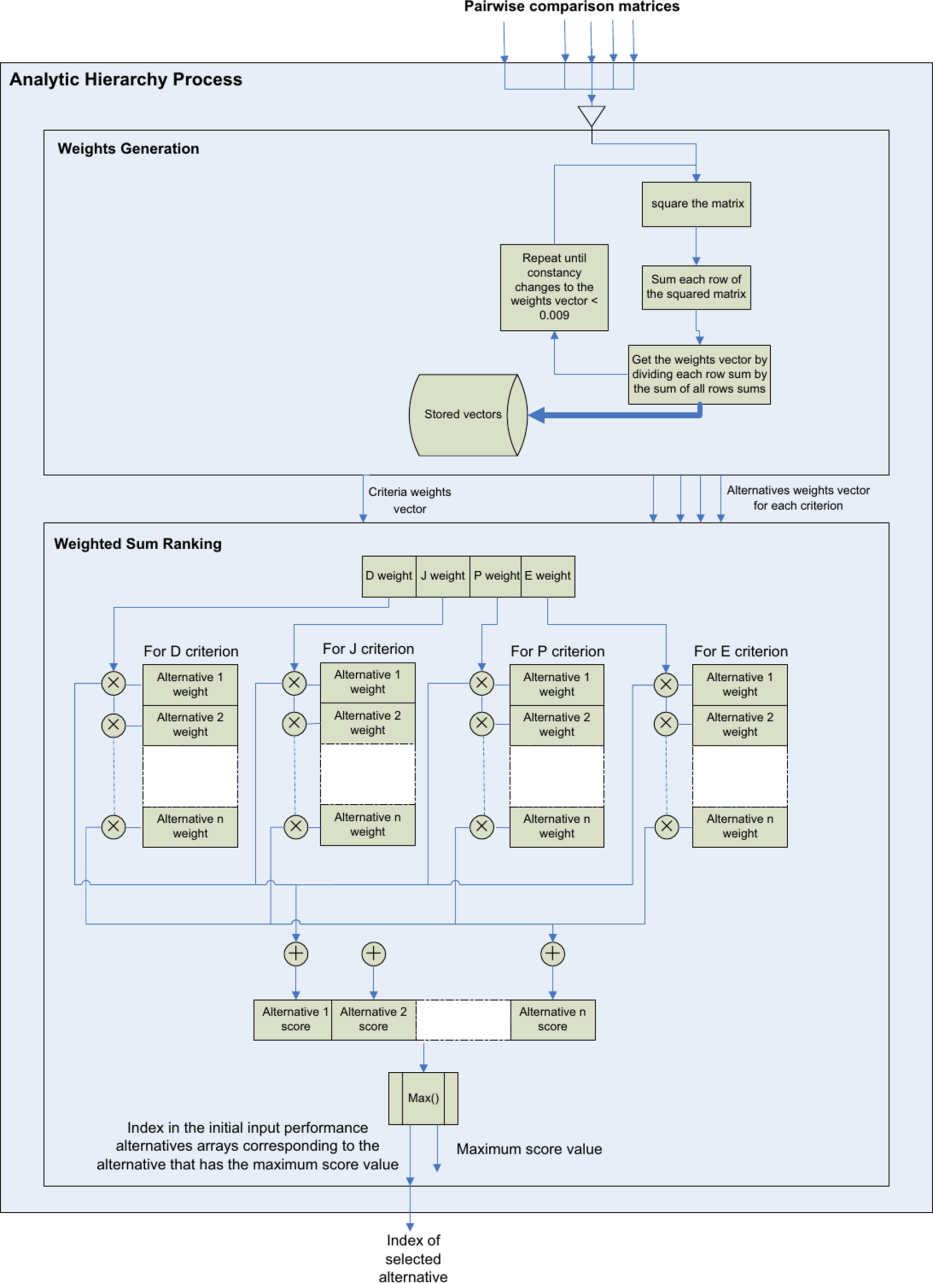
Represents the AHP stage of the analytical decision model proposed for parametric analysis.

The AHP stage, as shown in Fig. 8 takes the five pairwise comparison matrices and calculates the weights vectors and uses the weighted-sum model to compute alternatives’ scores, hence selecting the index of the best performance alternative.

The suggested values of all these tested parameters together with the determined best operating conditions form the recommended operating environment FAODVN-OE.

Under this recommended operating environment, a set of experiments were done employing the HELLO mechanism to test whether it will further improve the network performance.

Based on FAODVN-OE, amendments on the protocol can be proposed to improve the performance. An example of amendments to this base has been made and evaluated against the default AODV with the standard HELLO mechanism under the recommended operating conditions.

## Performance assessment experiments

This section describes the simulation experiments that were conducted to test FANET performance against changing conditions and parameters of AODV, tune the protocol, and propose modifications.

### The network model and assumptions

The assessed network consists of *N* drones flying outdoors in a 3D cubic space with dimensions *X, Y, Z*. There is only one ground base station (GBS) fixed in a known position on the ground. The drones continuously send data to GBS at different transmission intervals. It is assumed that:

- All drones are equipped with collision avoidance and positioning systems.

- For worst-case analysis, all the drones are assumed to be only mission-controlled, where each drone flies freely within the test space and decides its speed, direction, the time to change speed and direction independently of the others.

- The network employs only the source-initiated route repairing.

- The radio power consumption is the only considered when studying the energy consumption performance.

- The power consumption model used is based on the radio transceiver state where the energy consumption calculation is based on a constant power consumption of each state and the time spent on it.

**Fig. 9:**
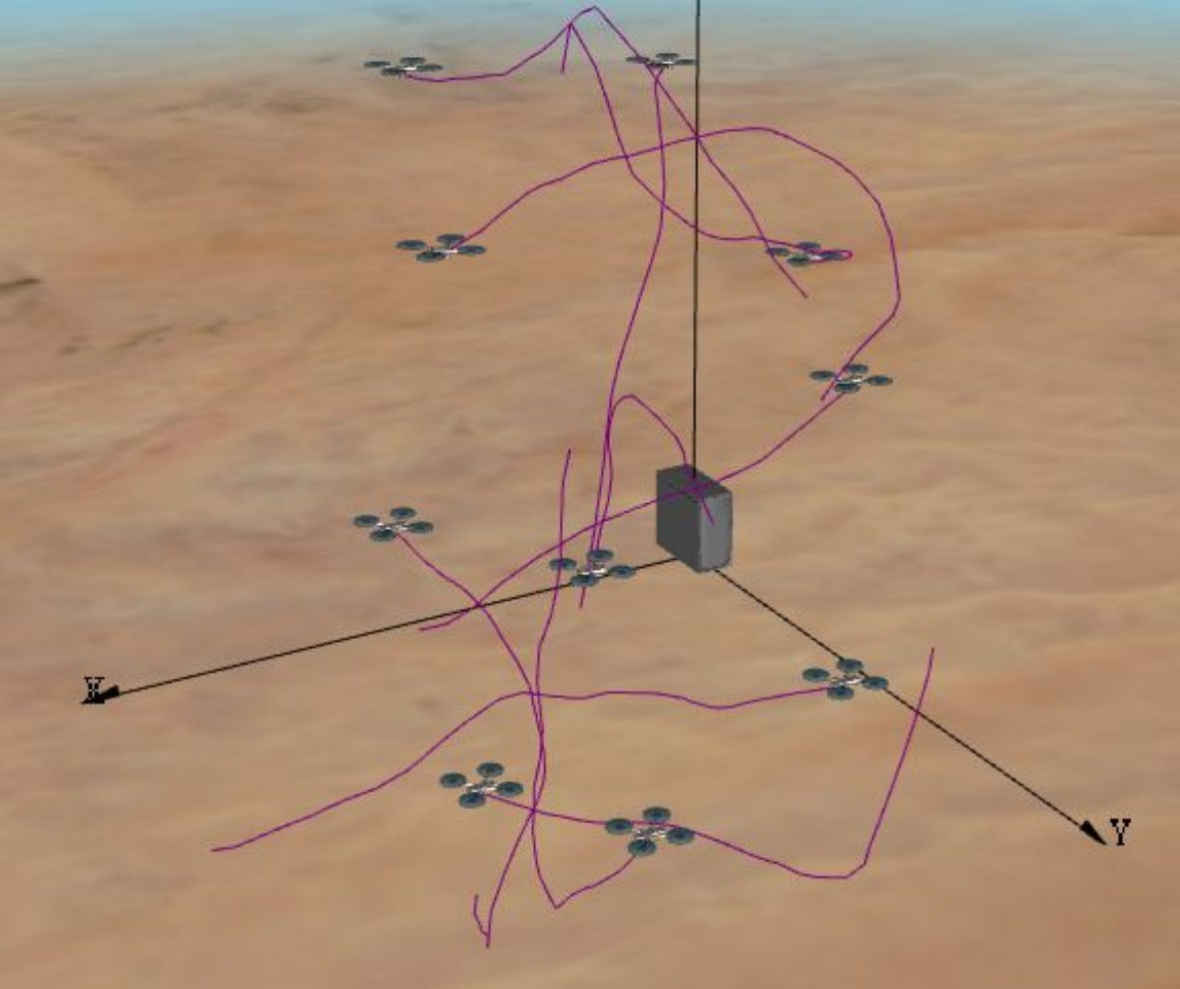
Snapshot of a simulated network. This a 3D view of a simulation run, where the simulation experiments were carried out using OMNeT +  + discrete-event simulator and the inet-4.2.5 framework.

A set of simulation runs were carried out in each experiment using the discrete event simulator OMNeT +  +^23^ and the inet-4.2.5 framework^24^. Each resulting value is the average of several runs. Table 1. shows the values of the key parameters and settings that were used in the experiments unless otherwise specified. Figure 9 shows a snapshot of a simulated network.

**Table 1:** Simulation parameters and settings.

Parameter	Value
*X, Y, Z*	1000, 1000, 1000 m
GBS position	(0, 0, 0)
Radio medium model	Apsk Scalar Radio Medium
Background noise power	−90 dBm
Sensitivity	−85 dBm
Antenna type	Constant gain antenna
Antenna gain	3 dB
Off-state power consumption	0 mW
Sleep-state power consumption	1 mW
Switching-state	1 mW
Idle-state	2 mW
Receiver Busy-state	5 mW
Receiving-state	10 mW
Transmitting-state	100 mW
MAC protocol	CSMA/CA-based with acknowledgements and retry mechanism
Acknowledgement Timeout	300 us
Bitrate	1 Mbps
Retry limit	7
MAC packet queue capacity	10
AODV protocol parameters	Default values
Frequency of changing speed and angle	1 Sec

### Performance metrics

Considering *U* as the set of all drones in the network, *P* is the set of all received data packets from all the drones at the GBS including the duplicate packets, $\acute{P}$ is the set of received data packets at the GBS from all the drones without duplicate packets, and *S* is the set of all data packets sent by all the drones.

$$P=\bigcup_{u\in U}{P}_{u,}$$

$$\acute{P}=\bigcup_{u\in U}\acute{P}_{u}$$

$$S=\bigcup_{u\in U}{S}_{u}.$$

I.e., the set of received data packets at the GBS equals the union of the sets of the received packets from each individual drone, and the set of sent data packets equals the union of the sets of the transmitted packets by each individual drone.

The performance of the network is evaluated through the following main metrics:

- The average end-to-end delay (${D}_{Avg}$) and it is computed in “Sec” from Eq. (1):

1 $$D_{Avg}=\frac{\sum_{p\in\acute{P}}D_{p}}{|\acute{P}|}=\frac{\sum_{p\in\acute{P}}(T_{r_p}-T_{c_p})}{|\acute{P}|}$$

where,${ D}_{p}$ is the end-to-end delay of a packet *p,*${T}_{{c}_{p}}$ is the creation time of the packet, and ${T}_{{r}_{p}}$ is the receiving time of the packet at the GBS.

- The Jitter which is the variation in end-to-end delay on a packet flow between a drone and the GBS. The average jitter (${J}_{Avg}$) is computed in “Sec” from Eq. (2):
  2 $${J}_{Avg}=\frac{\sum_{u=1}^{\left|U\right|}{\sum }_{p=1}^{\left|\acute{{P}_{u}}\right|}{J}_{u,p}}{\left|\acute{P}\right|}= \frac{\sum_{u=1}^{\left|U\right|}\left({ J}_{u,1}+( {\sum }_{p=2}^{\left|\acute{{P}_{u}}\right|}{J}_{u,p-1}+\frac{\left|{D}_{u,p}-{D}_{u,p-1}\right|-{J}_{u,p-1}}{16} )\right)}{\left|\acute{P}\right|}$$
  where,

${J}_{u,p}$ is the jitter of a packet *p* of a drone *u,*

${D}_{u,p}$ is the end-to-end delay of a packet *p* of a drone *u,*

$${J}_{u,1}=0      \forall u\in U$$

- The average route finding time (${T}_{Discov}$) and it is computed from Eq. (3):
  3 $${T}_{Discov}=\frac{{\sum }_{n=1}^{{N}_{Discov}}{{T}_{e}}_{n}-{{T}_{s}}_{n}}{{N}_{Discov}}$$
  where,

${N}_{Discov}$ is the number of all route discovery processes conducted by all the drones during mission lifetime,

${T}_{s}$ is the start time of a route discovery process,

${T}_{e}$ is the end time of a route discovery process.

- The throughput (*THR*) and it is computed in "byte/s" from Eq. (4):

4 $$\text{THR}=\frac{\sum_{p\in \acute{P}}{l}_{p}}{{T}_{sim}}$$

where, ${l}_{p}$ is the data payload length of a packet *p* in “byte” and ${T}_{sim}$ is the simulation duration in “Sec”.

- The packet delivery ratio (*PDR*) is computed from Eq. (5):

5 $$PDR= \frac{\left|\acute{P}\right|}{\left|S\right|}$$

- Average energy consumption (${E}_{Avg}$) and it is computed in “Joule” from Eq. (6):
  6 $${E}_{Avg}= \frac{\sum_{u\in U}{E}_{u}}{\left|U\right|}$$
  where, ${E}_{u}$ is the amount of energy consumed by a drone* u* at the end of the simulation.

### Experimental scenarios

Following our methodology, the experiments conducted fall under two main scenarios representing simulating AODV network with and without using HELLO messages. This means that the first assessment scenario deals with the case where sending redundant control messages for maintaining local connectivity is dispensed with, whereas the second scenario considers the effect of the optional HELLO mechanism meanwhile considers the proposal of modifications to the mechanism and generally to the protocol.

Nine experiments were carried out. The first three experiments are behavior observation experiments where the behavior of applying AODV in FANET are observed against increasing drone density, speed, and transmission interval, to conclude the best network conditions for applying AODV.

The second three experiments are parameters’ values testing experiments which are intended to test the effect of all possible values of AODV parameters rather than using their default values. The next two experiments are related to the second scenario. One of them is a significance testing experiment performed after tuning AODV FANET to test if there is still a need for maintaining local connectivity using the HELLO mechanism employed in AODV standard; the second one is a modification experiment where some simple possible modifications related to the HELLO mechanism and other procedures were suggested based on the principle that upon the optimized conditional parametric base for AODV-based FANET that was built throughout the previous experiments, the modifications to the standard design can be made to improve the performance. Eventually, the last experiment tests the recommended FAODVN-OE against the Default Values.

## Results and discussion

This section presents and discusses the results of implementing the simulated network model and performing the aforementioned experiments.

### Experiment1: AODV FANET behavior against increasing the drone density

**Fig. 10:**
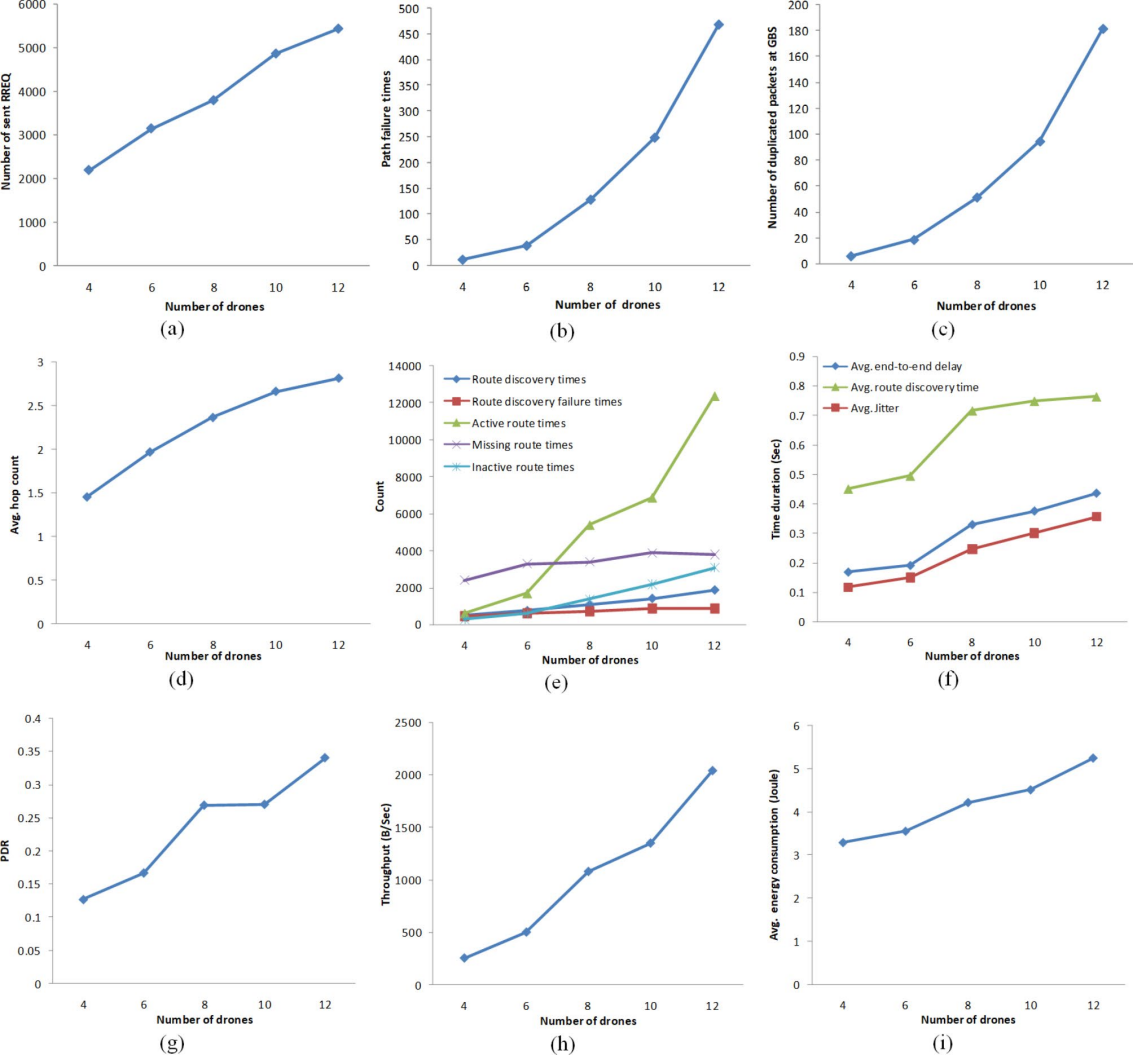
AODV-based FANET behavior against increasing drone density. (a) represents the number of sent RREQ messages against increasing the number of flying drones, (b) represents the number of times a discovered path has been failed against increasing the number drones, (c) represents the number of duplicated packets received at the GBS against increasing the number drones, (d) represents the average count of hops constructing the discovered paths, (e) represents important counts related to the path discovery process against increasing the number drones: route discoveries, route discovery failures, times of canceled discovery due to an existing active route found, times of an originator node found no existing route, and times of an originator node found an existing route but in an inactive state, (f) represents delay-related parameters against increasing the number drones, (g) represents the packet delivery ratio against increasing the number drones, (h) represents the throughput against increasing the number drones, and (i) represents the average energy consumption per drone against increasing the number drones.

Taking the speed range of drones as (5 m/s-10 m/s) and the transmission interval is exponentially distributed with mean 2 Sec, the network density is increased by increasing the number of deployed drones from 4 to 12 with step 2. Figure 10 shows the obtained results. As indicated in Fig. 10, the increased density caused congestion, the number of data packets and RREQ sent through the network are increased linearly, the number of times a routing path failed is increased with polynomial growth as well as the number of duplicated packets received by the GBS and the average hop count. This causes the route finding time and the end-to-end delay to increase with density.

The average route finding time is greater than the average end-to-end delay by 124%, accordingly, the variation in the end-to-end delay has a big value, as shown in Fig. 10. the average jitter is about 77.4% of the average end-to-end delay.

Figure (10-e) shows the behavior of different counts: the count of the route discovery processes incurred by all drones, the count of failure times of this process, counts related to the case when a data packet is about to be transmitted whether generated or forwarded indicate the number of times an active route to the GBS is found, the number of times the route to the GBS is missed, and the number of times a route to the GBS is found but it is inactive. As indicated in Fig. (10-e), when the drones density is small, the count of the missed data routes is greater than the found active routes; as the density increases, the two counts increases but the missed routes count increases with slower growth rate. Also noteworthy is the increase in the count of the found inactive routes with smaller growth than the found active routes. When the number of deployed drones is 12, the number of found active routes is greater than found inactive and missing routes by 303.5% and 226%, respectively.

The number of route discoveries increases linearly with the linear increase in the sum of the missing and inactive routes but with smaller slope as not each route assessed to be missing or inactive resulted in a new discovery process if another one is ongoing. In other words, the route failure causes the number of route discoveries to increase, while the increase of discovery duration reduces it.

Although the increase in density causes increase in the network congestion and overhead, increase in path failure, logarithmic increase in route discovery failure with increase in the discovery time, Fig. (10g) and Fig. (10h) indicate that the PDR and the throughput increase with the increase in density.

From the obtained behavior, it could be concluded that the increase in drones density causes slight increase in routes failure and route discovery failure, at the same time causes frequent use of drones and paths which helps AODV FANET to produce extended lifetime routes and accordingly increases the chance of an active route for each message to be sent. This benefits the network to produce increased PDR and throughput, but at the expense of higher energy consumption and end-to-end delay. The big value of route discovery time results in big value of jitter.

Controlling the route discovery process by decreasing the count of discoveries and the duration of discovery, and controlling the start time of the discovery such that it ends before the start of data packet sending would overcome the mentioned degradation of AODV FANET performance. Also, the count of failure times of the route discovery process can be improved while considering the other performance metric trade-offs by controlling the route reply mechanism including the binary exponential backoff time waiting for a route reply and the value of RREQ_RETRIES.

### Experiment2: AODV FANET behavior against increasing the speed of drones

**Fig. 11:**
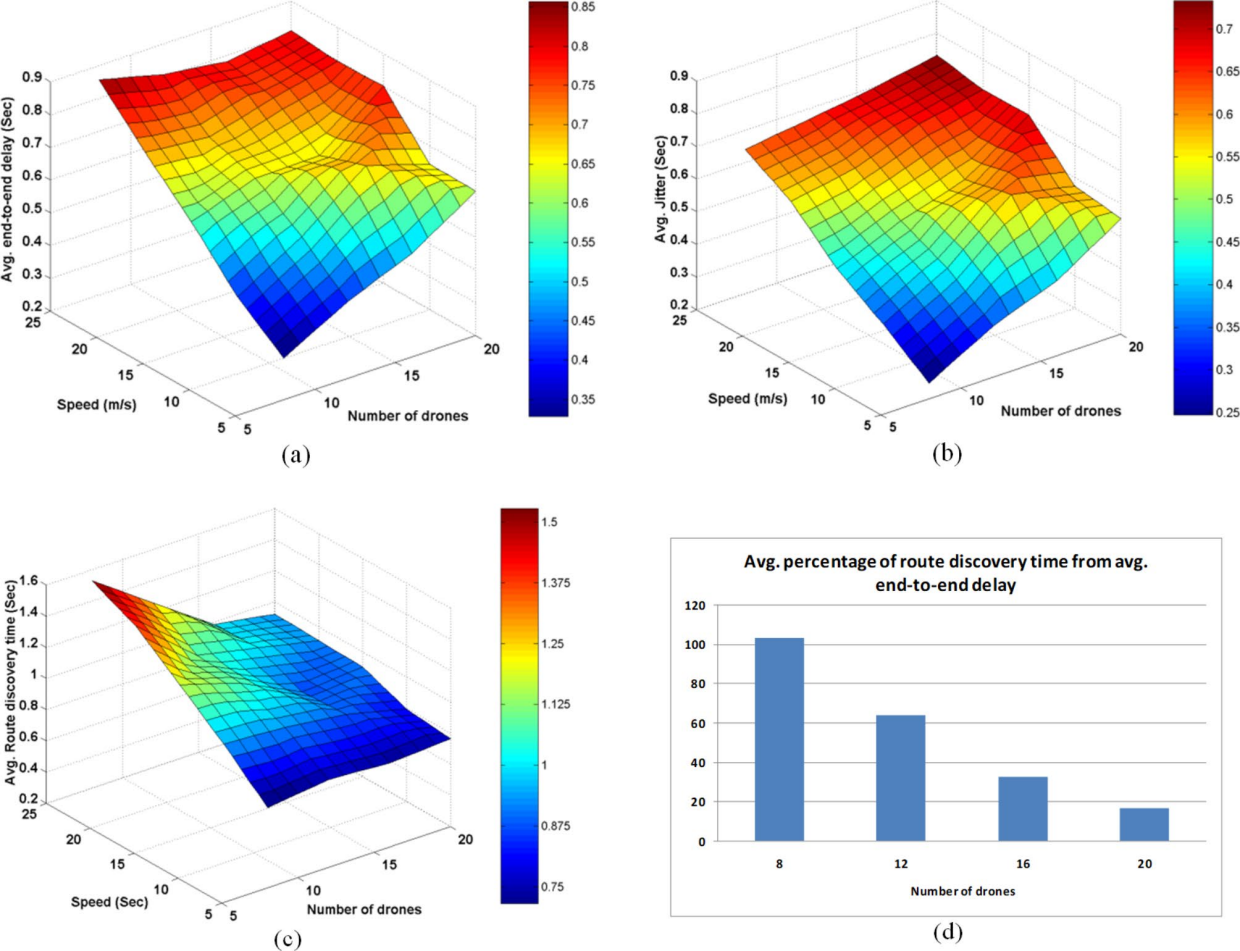
AODV-based FANET behavior of time-related parameters against increasing drone speed and density. (a) represents the average end-to-end delay parameter, (b) represents the average jitter parameter, (c) represents the average duration of the performed discovery processes, and (d) shows the percentage of the average value of the route discovery duration from the average value end-to-end delay at different drone densities.

**Fig. 12:**
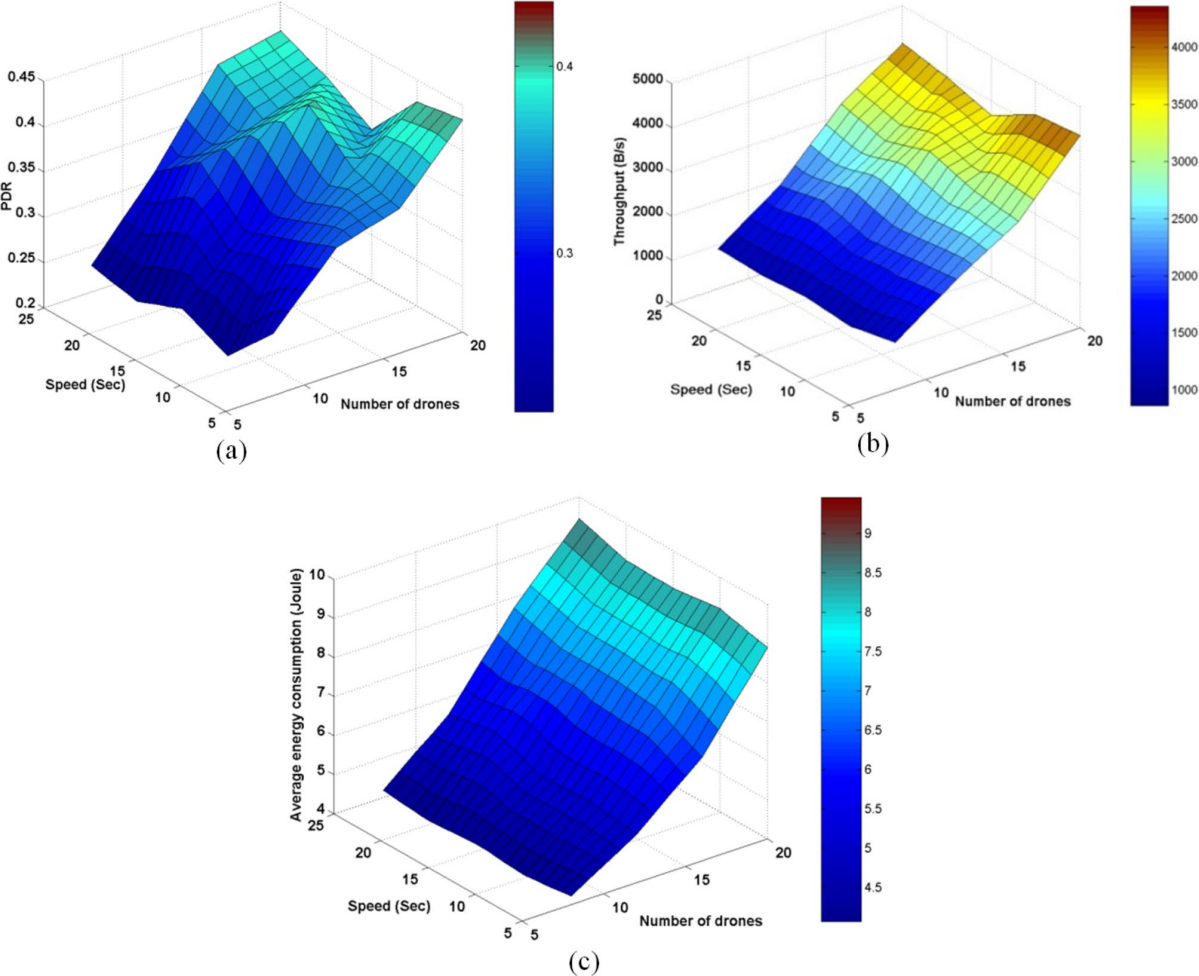
AODV-based FANET performance metrics against increasing drone speed and density. (a) represents the packet delivery ratio, (b) represents the throughput parameter, and (c) represents the average energy consumption parameter.

**Fig. 13:**
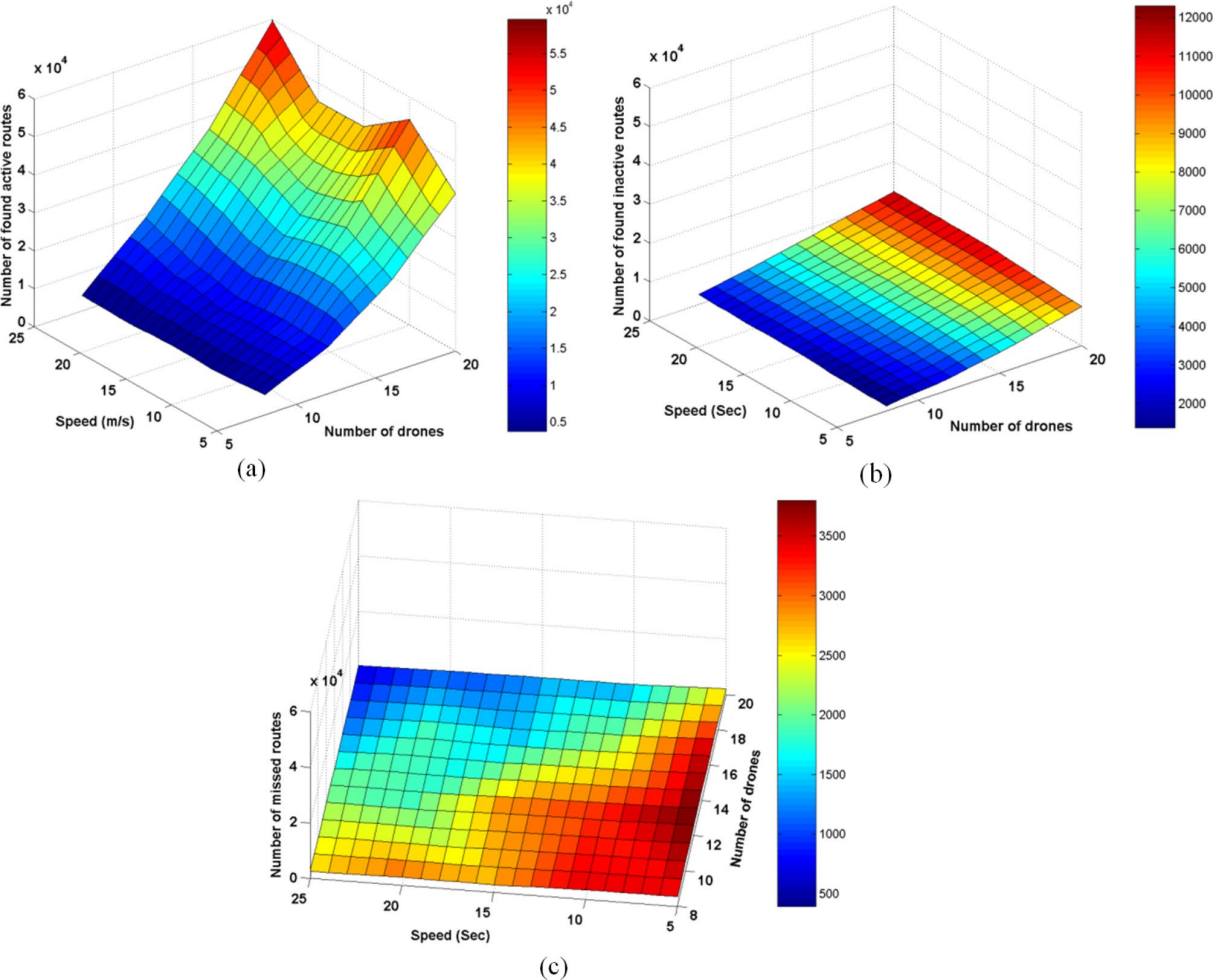
AODV-based FANET behavior of different counts against increasing drone speed and density. (a) represents times of canceled discovery due to an existing active route found, (b) represents times of an originator node found an existing route but in an inactive state, and (c) represents times of an originator node found no existing route.

In this experiment, the speed range of drones is increased to take the following ranges: (5 m/s-10 m/s), (10 m/s−15 m/s), (15 m/s−20 m/s), (20 m/s−25 m/s), and (25 m/s−30 m/s), and the behavior of the network is observed for both of these numbers of drones: 8, 12, 16, and 20; the results are depicted in Figs. 11, 12, 13.

The increase in drones speed didn’t affect some parameters such as the average hop count. The number of RREQ increases with negligible amount, and the number of route discovery failures decreases with negligible amount. The number of route discovery processes slightly increases at the higher speeds and higher number of drones. The number of routing paths failure increases with the increase in speed, and this is more apparent at the higher drones number.

Form Fig. 11, it could be concluded that using smaller number of drones in AODV-based FANET at lower speed gives better performance in terms of the average end-to-end delay, while in higher speeds, more than 25 m/s using larger number of drones gives better performance.

The discovery duration increases linearly with the increase in speed, but the slope of the curve decreases with the increase in drones number, this appears in Fig. (11-c) and Fig. (11-d) where the percentage by which the discovery time is greater than the average end-to-end delay decreases with the increase in speed. Also, it is worth noting that the discovery duration is found to be decreased with the increase in drones at the higher speeds this in contrast to the finding of Experiment1 under lower speeds condition.

The average jitter increases with the increase in drones’ number and speed, and its percentage from the average end-to-end delay is still big and increases slightly with the increase in speed.

As observed from Experiment1, the number of found active routes increases with the increase in drones number, and increases more at higher speeds, but the lower number of drones, less than ten, produces decaying curve with respect to the drones speed. As the number of found inactive routes increases with drones number, it increases with speed. By increasing the drones number more in this test, it is found that the number of missed routes decreases with speed as well as with nodes number.

It could be said that the PDR, throughput, and average energy consumption didn’t affected by the speed increase though average energy consumption increased slightly at higher speeds.

It could be concluded from the above findings that AODV-based FANET gives better performance at higher drone density and low speeds, however it needs improvements in the end-to-end delay and energy consumption without affecting the other metrics. The higher density results in the best PDR and throughput at the expense of energy consumption, and in this case, the low speeds gives the better performance in terms of average end-to-end delay and jitter.

To more comprehensively determine the conditions under which the default AODV-based FANET gives the best performance, we need also to consider the rate at which the drones send data, thus this will be addressed in the following section.

### Experiment3: AODV FANET behavior against increasing the transmission interval

In this experiment, the performance of the network is tested over different ranges of the transmission interval at the conditions of low and high speeds at low and higher value of drone density. The different ranges of the transmission interval are: (0.5 Sec−3 Sec), (5 Sec−10 Sec), (20 Sec−60 Sec), and (120 Sec−300 Sec). A slightly different transmission pattern more suitable to application scenario followed previously in this paper is employed in this experiment. Previously the drone was changing continuously the interval after which it transmits its data, while in this experiment, each drone starts to transmit data at different time uniformly distributed over the range (1 Sec−20 Sec), and then follows a constant transmission interval picked randomly from a uniform distribution represents one of the four ranges stated before.

**Fig. 14:**
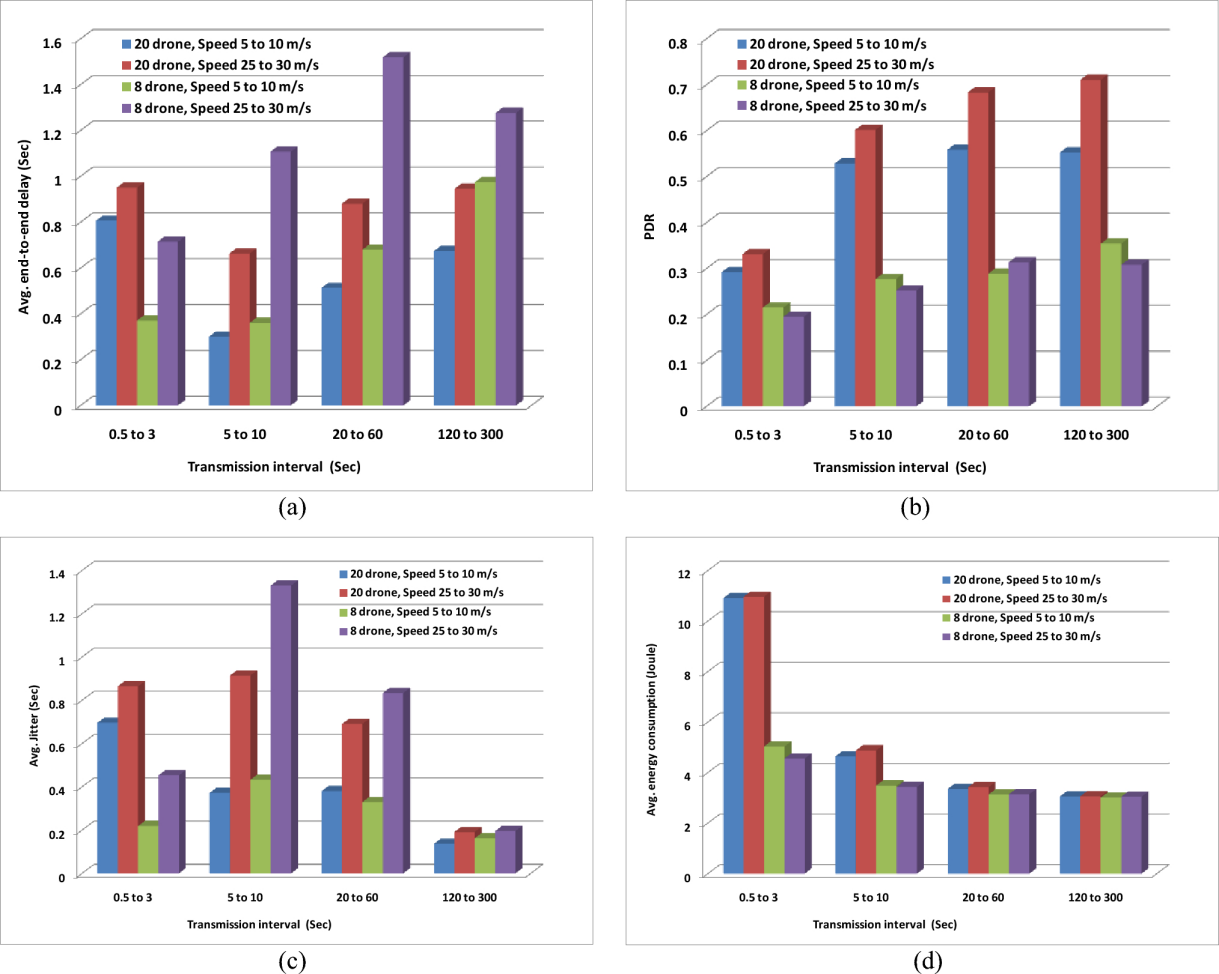
AODV-based FANET behavior against increasing drone speed, density, and transmission interval. This figure assesses the performance of the network under different operating conditions: the number of flying drones, and the transmission interval and speed of each drone, (a) represents the performance with respect to average end-to-end delay, (b) represents the performance with respect to packet delivery ratio, (c) represents the performance with respect to average jitter, and (d) represents the performance with respect to average energy consumption.

Figure 14 shows the experiment results, and Table 2 indicates the preferred and undesirable cases. Any case incorporates high density and high rate should be avoided as it results in high energy consumption while doesn’t give good performance with respect to the other metrics.

**Table 2:**
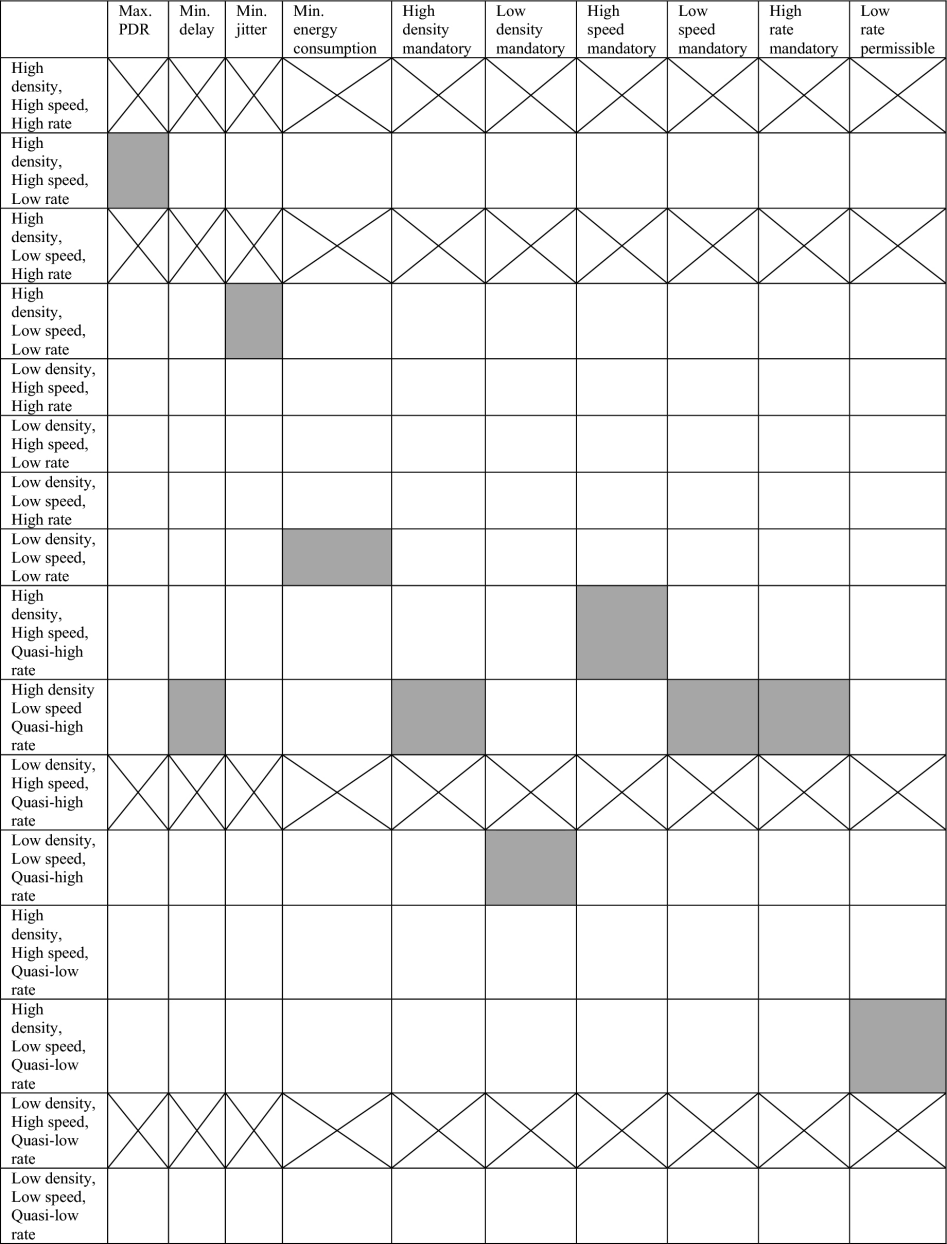
Analysis of best performance conditions of default AODV-based FANET: drone density, drones speed, and drones transmission rate*

*Quasi-low rate means 20-60 Sec transmission interval and quasi-high rate means 5-10 Sec transmission interval. Grey-shaded cells are the preferred. The cross cells refer to the cases that should be avoided as stated in this section.

According to Cisco press, for real-time communication, a delay of maximum 0.3 Sec results in smooth communication, and jitter of maximum 0.03 Sec gives good user experience.

The only case which satisfies this delay limit is the case of High density, Low speed, and Quasi-high rate, while there is no case satisfies the jitter limit which entails adjustments to improve the jitter of the AODV FANET with default parameters to be suitable for conveying audio and video.

Two other cases should be avoided which are Low density with High speed under Quasi-high or Quasi-low rate where they produce high delay and jitter while the resulted PDR value is small.

Based on a notice from Table 2, it could be said that the case of High density, Low speed, and Quasi-high rate is the most preferred one for the default AODV FANET as it satisfies the delay limit, and other metrics may be further improved by adjusting AODV parameters.

The cases that appear in Table 2 which are not indicated as preferred or undesirable can be used to tune the performance at run time.

### Experiment4: Testing three key AODV parameters

**Fig. 15:**
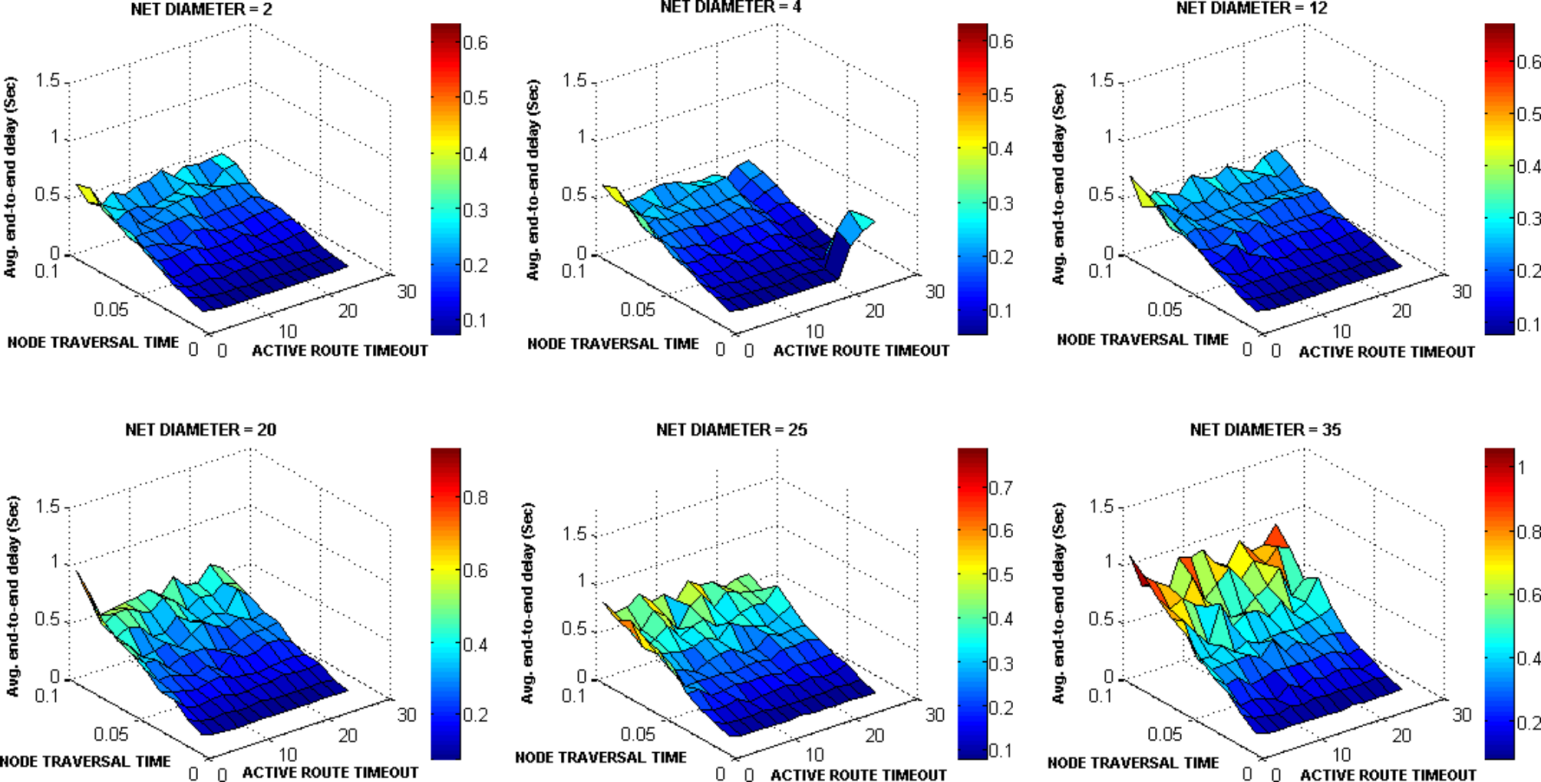
AODV-based FANET end-to-end delay performance under different parameters values and preferred operating conditions.

**Fig. 16:**
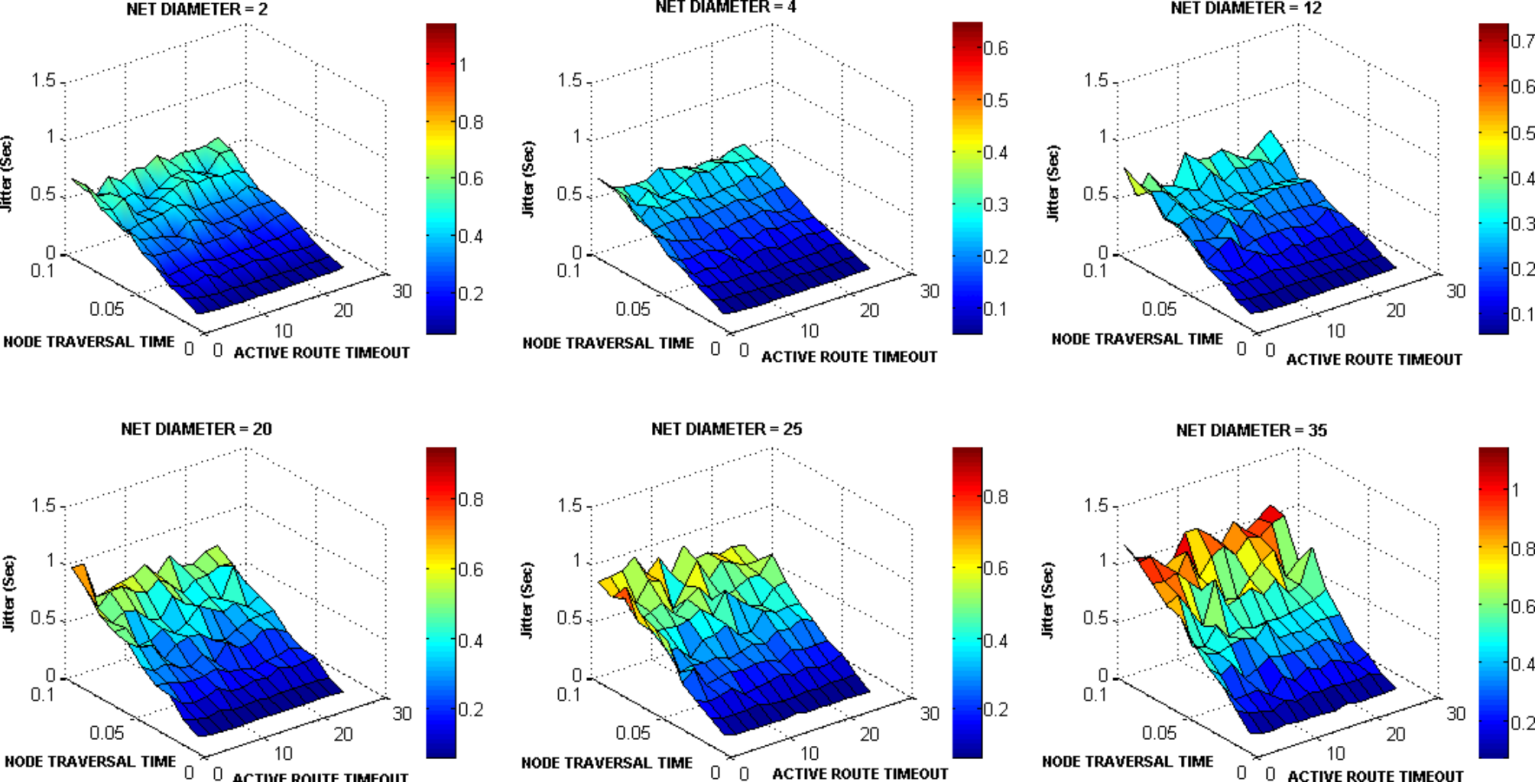
AODV-based FANET jitter performance under different parameters values and preferred operating conditions.

**Fig. 17:**
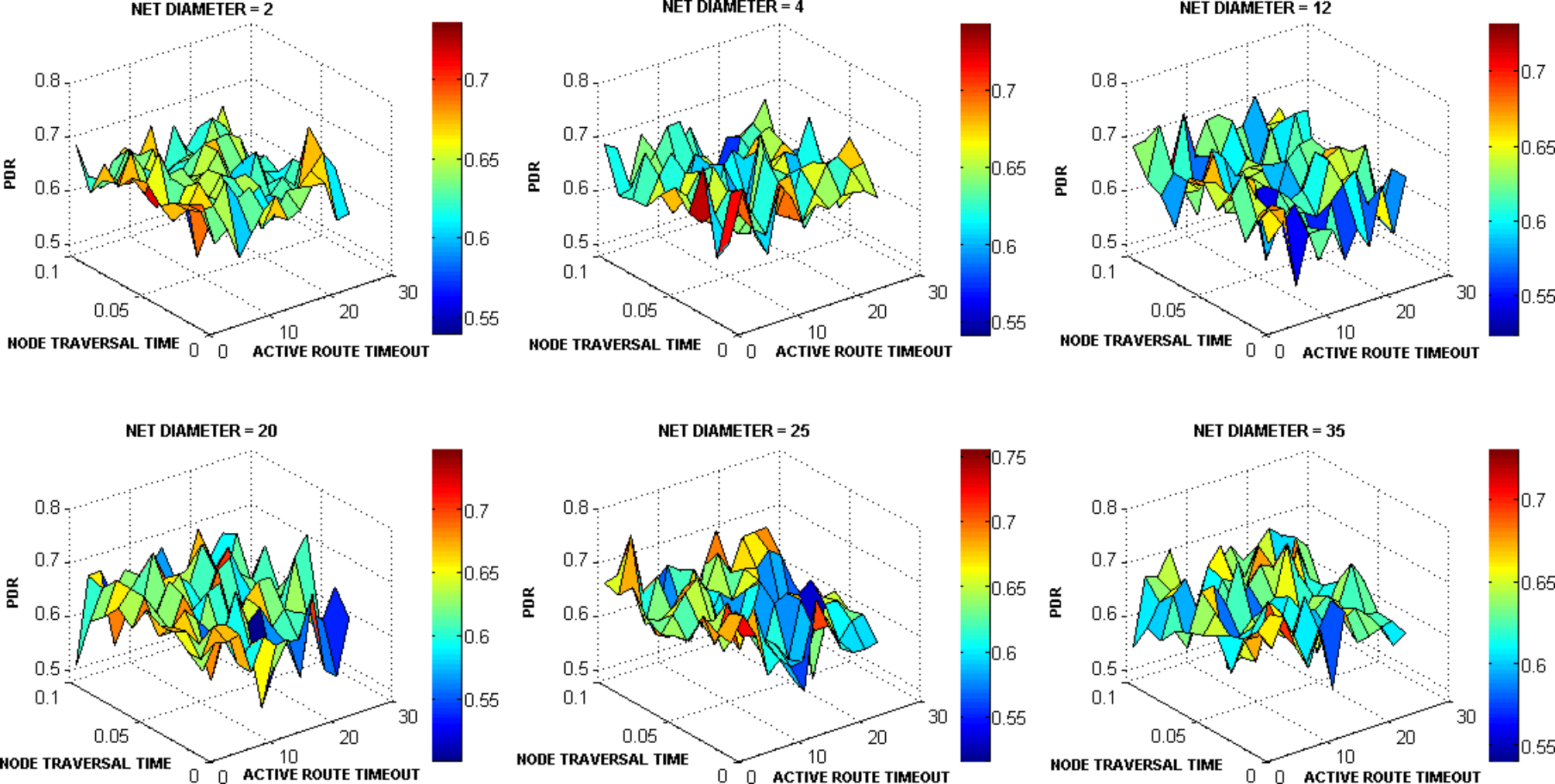
AODV-based FANET PDR performance against NODE_TRAVERSAL_TIME values under different parameters values and preferred operating environment.

**Fig. 18:**
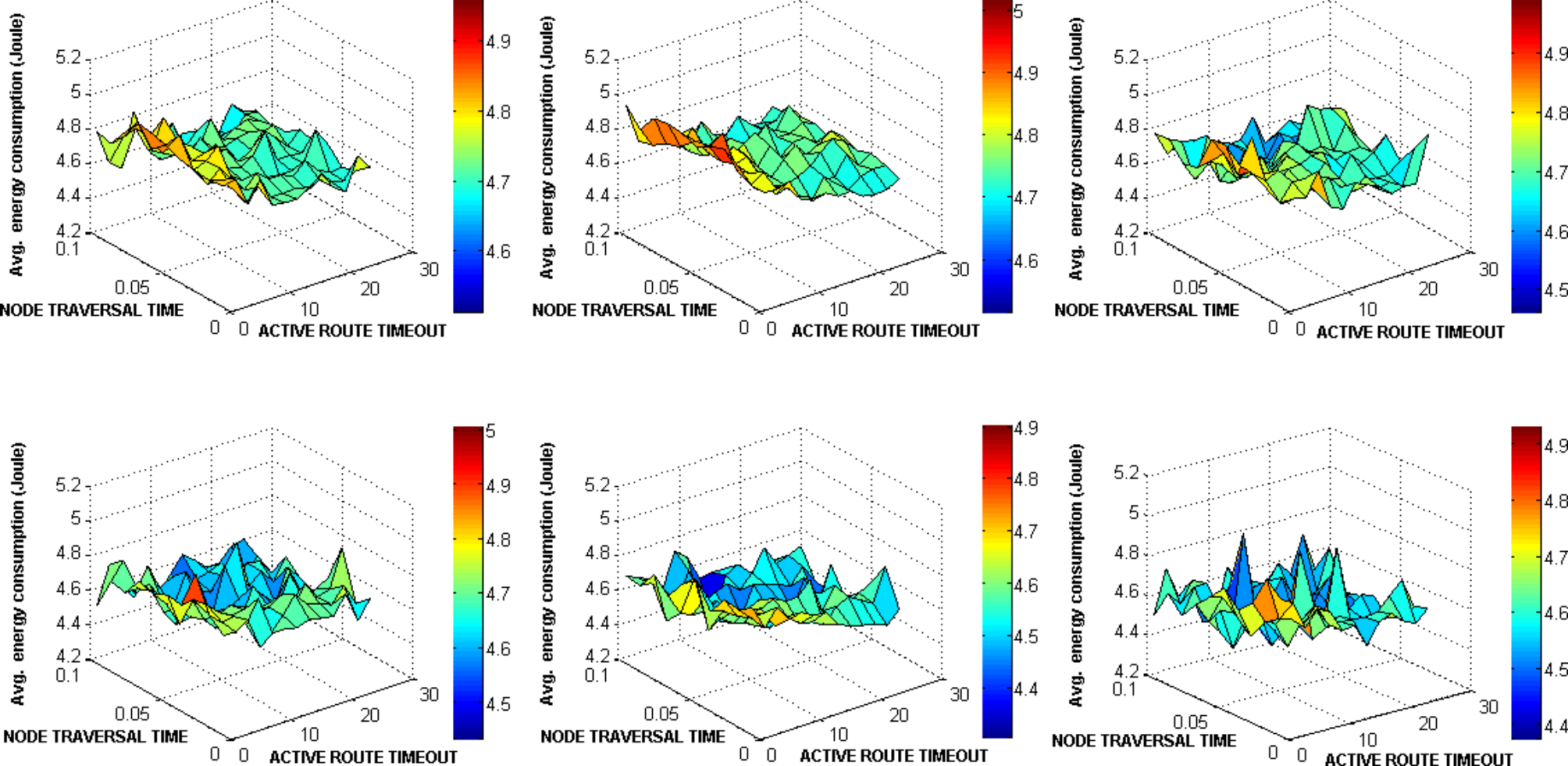
AODV-based FANET energy consumption performance against NODE_TRAVERSAL_TIME values under different parameters values and preferred operating environment.

The ACTIVE_ROUTE_TIMEOUT, NET_DIAMETER, and NODE_TRAVERSAL_TIME were selected first for testing as a lot of other AODV parameters are functions of them. The ACTIVE_ROUTE_TIMEOUT value was increased from 1 to 25 Sec with step 2. The NODE_TRAVERSAL_TIME value was increased from 0.01 Sec to 0.1 Sec. Different values of NET_DIAMETER were employed {2, 4, 12, 20, 25, 35}. The value 4 expresses the average hop count and value 2 expresses a smaller value. The value 20 represents the actual maximum possible number of hops which equals the number of drones, and the value 35 is the AODV default value. The values 12 and 25 represent intermediate values. Figures 15, 16, 17, 18 graph the output of testing the different combinations of these three parameters.

By analyzing the output, the jitter is smaller than delay at lower NODE_TRAVERSAL_TIME values, and the difference between them decreases with increasing NODE_TRAVERSAL_TIME until the jitter becomes greater than the delay at higher values of NODE_TRAVERSAL_TIME at any value of NET_DIAMETER, and both of their values increase with the increase in NODE_TRAVERSAL_TIME. The 0.3 Sec limit of delay is satisfied only at low NODE_TRAVERSAL_TIME values. The curves of delay and jitter with ACTIVE_ROUTE_TIMEOUT have negative slope approaches zero at high NODE_TRAVERSAL_TIME values. Therefore high values of NODE_TRAVERSAL_TIME is not recommended with respect to the delay and jitter performance. The increase of NET_DIAMETER has a negative effect on delay and jitter at low NODE_TRAVERSAL_TIME values.

The general trend of energy consumption curve versus ACTIVE_ROUTE_TIMEOUT is decay, and it could be said that the smaller values of energy consumption occurs at higher values of the three parameters.

The PDR tends to decrease with the increase in ACTIVE_ROUTE_TIMEOUT by small slope at low NODE_TRAVERSAL_TIME values; this tendency turns into constant relationship at high NODE_TRAVERSAL_TIME and low NET_DIAMETER, and increasing curve at high NODE_TRAVERSAL_TIME and NET_DIAMETER values.

In general, the variation in PDR values is small; the standard deviation of PDR values is about 0.04 which is less than the standard deviation of the other metrics values by more than 64%, and PDR has weaker correlation with the NODE_TRAVERSAL_TIME and NET_DIAMETER of correlation coefficients -0.065 and -0.029, respectively. Using the decision model for parameters values selection followed in our methodology, it was found that the values of NET_DIAMETER, ACTIVE_ROUTE_TIMEOUT, and NODE_TRAVERSAL_TIME which give the best performance are 2, 13, and 0.01, respectively, corresponding to delay, jitter, PDR, and energy consumption values of 0.0738145 Sec, 0.056167 Sec, 0.675, and 4.6534351 Joule, respectively.

### Experiment5: Testing RREQ dissemination control parameters

The focus of this experiment is the effect of the parameters related to the expanding ring search process for RREQ dissemination. The values of TTL_START, TTL_THRESHOLD, and TTL_INCREMENT were varied to test their combinations effect; TIMEOUT_BUFFER kept as the default value.

Rationally, the value of TTL_START should be less than or equal the NET_DIAMETER value, and the normal behavior of the expanding ring search is achieved when TTL_THRESHOLD is less than or equal the NET_DIAMETER. In this case, the behavior of the network and accordingly its performance is divided into intervals corresponding to intervals of TTL_THRESHOLD values, over each interval the behavior remains constant. The TTL_THRESHOLD intervals are:

$$\left[{\text{THRSH}}_{\text{min}} ,\text{START}+\text{INCREMENT}\right],$$

$$\left(\text{START}+\text{INCREMENT },\text{ START}+2\times \text{INCREMENT }],\right.$$

$$\left(\text{START}+2\times \text{INCREMENT },\text{ START}+3\times \text{INCREMENT }],\dots \dots \dots .,\right.$$

$$\left(\text{START}+n\times \text{INCREMENT }, {\text{THRSH}}_{\text{max}} ]\right.$$

where $$n= \lceil\frac{{\text{THRSH}}_{\text{max}}-\text{START}}{\text{INCREMENT }}-1\rceil$$

**Fig. 19:**
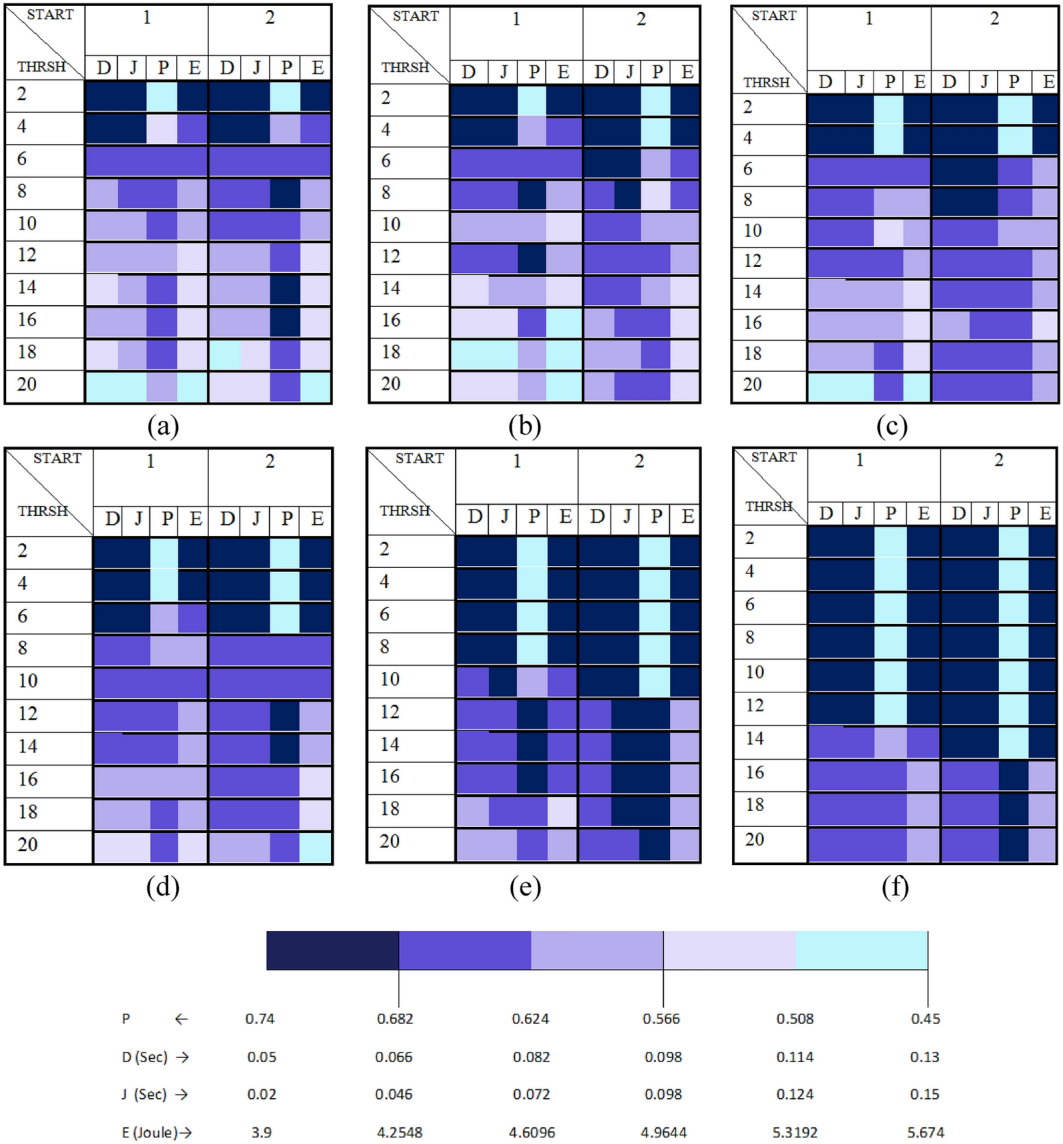
AODV-based FANET performance under different values of expanding ring search process parameters, P stands for PDR, D stands for delay, J stands for jitter, and E stands for energy consumption, (a) TTL_INCREMENT = 1, (b) TTL_INCREMENT = 2, (c) TTL_INCREMENT = 3, (d) TTL_INCREMENT = 4, (e) TTL_INCREMENT = 8, (f) TTL_INCREMENT = 12.

According to the selected NET_DIAMETER value, only the values 1 and 2 for TTL_START were considered in testing, while the effect of using a wider range of TTL_THRESHOLD values were tested as indicated in Fig. 19.

Taking the NET_DIAMETER less than the TTL_THRESHOLD causing the system doesn’t enter the steady state that exists when the TTL value remains unchanged at NET_DIAMETER, but it causes loops in TTL value between the NET_DIAMETER and the TTL_THRESHOLD which may change the mentioned TTL_THRESHOLD intervals depending on the TTL_START value.

Referring to Fig. 19, according to what the scale of the colorbar of the heatmap charts is interpreting, the best performance is corresponding to the cell with the largest dark blue area.

No configuration of the conducted experiments achieves the best performance with respect to all the metrics; no configuration achieves more than 75% of dark blue area. There is a clear trade-off between the PDR and the other metrics.

It can be concluded from Fig. 19 that the performance of AODV FANET is better at $\text{THRSH }\in \left[{\text{THRSH}}_{\text{min}} ,\text{START}+\text{INCREMENT}\right]$ at any value of $\text{INCREMENT}$.

The decision model for parameters values selection was used to find the configuration which gives the best performance, and it was found that more than one configuration gives the same best performance corresponding to TTL_START = 2 and any TTL_INCREMENT value at $\text{TTL}\_\text{THRESHOLD }\in \left[{\text{THRSH}}_{\text{min}} ,\text{START}+\text{INCREMENT}\right]$, where the achieved performance satisfies both the delay and jitter limits.

### Experiment6: Controlling the number of route discovery attempts

**Fig. 20:**
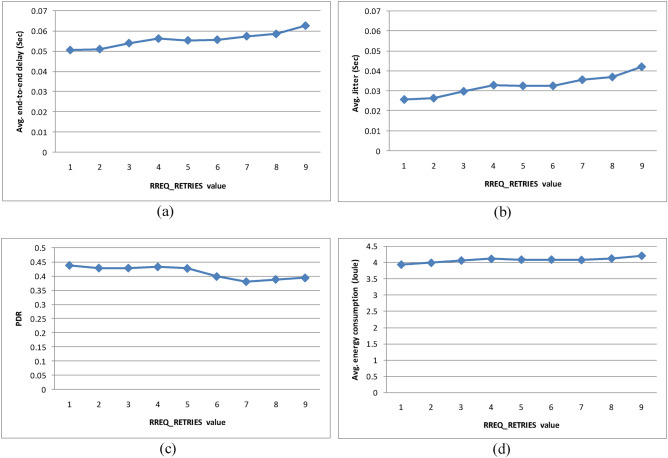
AODV-based FANET performance under different RREQ_RETRIES values. (a) represents the performance with respect to average end-to-end delay, (c) represents the performance with respect to average jitter, (c) represents the performance with respect to packet delivery ratio, and (d) represents the performance with respect to average energy consumption.

This experiment builds on the previous selected AODV FANET settings, taking TTL_INCREMENT = 2 and $\text{TTL}\_\text{THRESHOLD}$ = 2, and tests the effect of different RREQ_RETRIES values. Figure 20 shows the results of increasing RREQ_RETRIES value from 1 to 9. The average end-to-end delay, the average jitter, and average energy consumption increases with the increase in RREQ_RETRIES value, while the PDR decreases. Visually and using the analytical decision model, it could be concluded that setting RREQ_RETRIES equals 1 gives the best performance.

### Experiment7: Testing HELLO mechanism significance

**Fig. 21:**
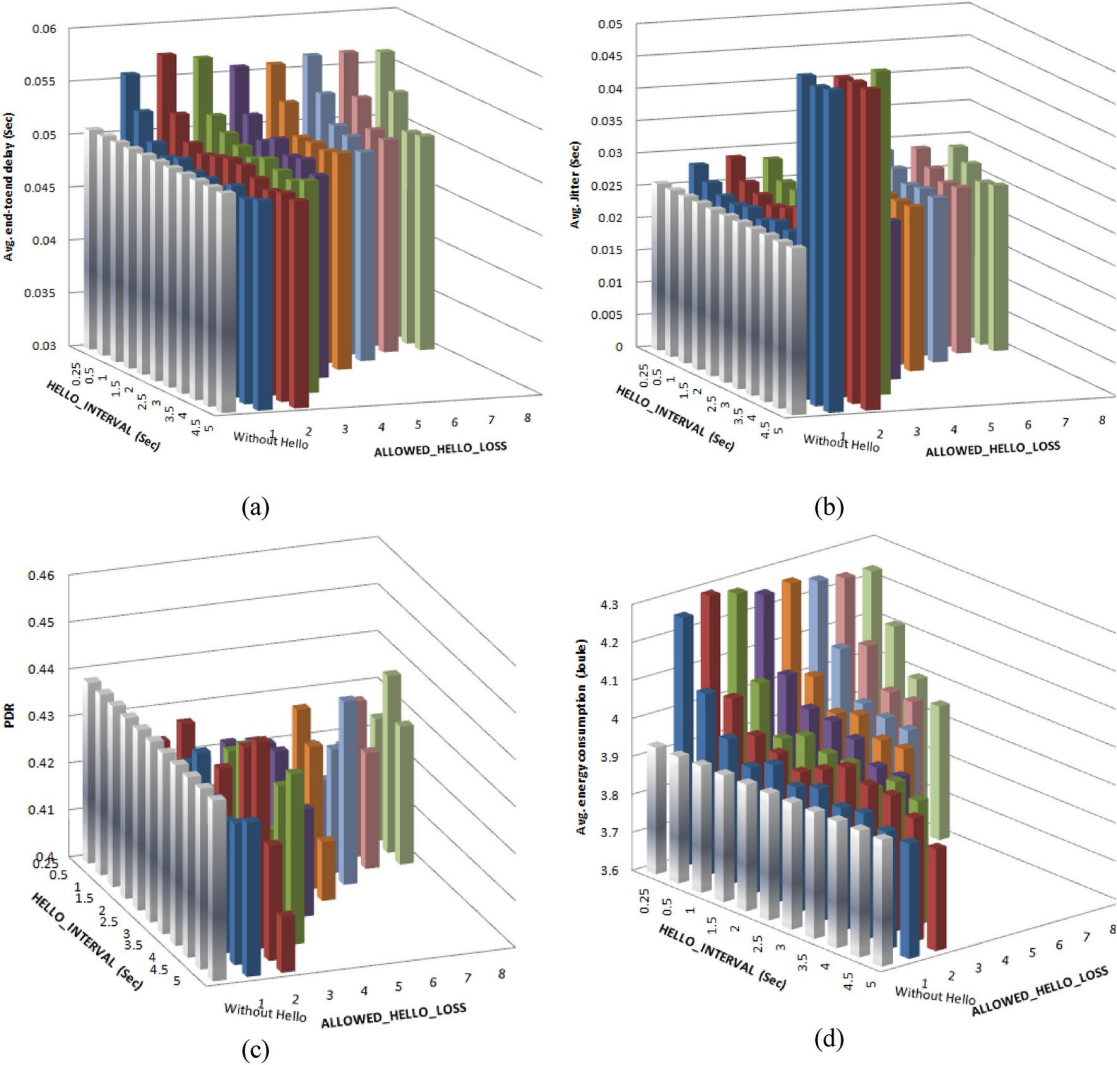
Comparing AODV-based FANET performance under the recommended operating environment without using Hello and with Hello. (a) represents the performance with respect to average end-to-end delay, (c) represents the performance with respect to average jitter, (c) represents the performance with respect to packet delivery ratio, and (d) represents the performance with respect to average energy consumption.

This experiment is performed after tuning AODV FANET to test if there is still a need for maintaining local connectivity using the HELLO mechanism employed in AODV standard. To perform this test, the two parameters that controlling this process, HELLO_INTERVAL and ALLOWED_HELLO_LOSS, were varied taking into account the condition that requires ACTIVE_ROUTE_TIMEOUT to be greater than the value ALLOWED_HELLO_LOSS × HELLO_INTERVAL; the results are depicted in Fig. 21 where the performance is compared to the result of the first scenario.

From the obtained results, it can be concluded that after adjusting the timing parameters and the RREQ dissemination parameters, the HELLO mechanism of AODV standard doesn’t improve more the network performance, the number of Hello messages that were not sent due to other broadcasts during the last HELLO_INTERVAL is on average 154% greater than the sent Hello messages, but it increases the network overhead and congestion, and accordingly degrades the overall performance slightly. However, the standard mechanism is subject to modifications to further improve performance.

### Experiment8: Modifying the standard HELLO mechanism

Upon the optimized conditional parametric base for AODV-based FANET that was built throughout the previous experiments, modifications can be made to the standard design to improve performance. In this experiment, some simple possible modifications related to the HELLO mechanism and other procedures will be suggested as described below,

- To decrease the number of Hello messages without affecting its functionality, it is better to send them only when data is expected to be transmitted. Also, they are needed to be sent in a time allows for a period enough for the drone that is expected to transmit or convey data to detect the loss of connection to the next hop, propagate notification, and a new path is discovered. Therefore, the conditions that are tested every HELLO_INTERVAL to determine whether to send Hello message or not were modified to include a new condition as follows:
  Assuming that the packet arrival to a drone follows a Poisson process with the average arrival rate as *λ* packet/s, the condition is:
  7 $$\text{P}\left(\text{at least one transmission occurs duirng }{T}_{det\_recov}\right)\ge 0.999$$
  8 $$\text{P}\left(\text{at least one transmission occurs duirng }{T}_{det\_recov}\right)=1-\text{P}(\text{no neighbor conveys data duirng }{T}_{det\_recov})$$
  9 $$\text{P}(\text{no neighbor conveys data duirng }{T}_{det\_recov}) =\prod_{i \in {S}_{n}}{\text{e}}^{-{\lambda }_{i}{T}_{det\_recov}}$$
  10 $${T}_{det\_recov}={N}_{Loss}\times {T}_{Interv}+2\times {H}_{Avg}\times {T}_{Trav}+{T}_{MaxDiscov}$$
  The arrival rate* λ* is computed over the past ${T}_{det\_recov}$ in terms of ${T}_{Interv}$ as a unit of time,
  11 $${\lambda }_{i}=\frac{\sum_{j=1}^{\lceil\frac{{T}_{det\_recov}}{{T}_{Interv}}\rceil}{NP}_{i,j}}{\lceil\frac{{T}_{det\_recov}}{{T}_{Interv}}\rceil\times {T}_{Interv}}$$
  where,
  ${S}_{n}$ is the set of all drones that have conveyed data to this drone and are currently part of an active route.
  ${T}_{det\_recov}$ is an estimation of the period of path failure detection and recovery before data transmission time.
  ${N}_{Loss}$ is the ALLOWED_HELLO_LOSS.
  ${T}_{Interv}$ is the HELLO_INTERVAL.
  ${H}_{Avg}$ is the average hop count.
  ${T}_{Trav}$ is the NODE_TRAVERSAL_TIME.
  ${T}_{MaxDiscov}$ is the maximum path discovery time.
  ${NP}_{i,j}$ is the number of data packets received from a drone *i* in a time interval *j* equals to the unit of time.
- In the employed network scenario, there is only one destination, and the data is consciously conveyed to it by all drones, therefore to decrease the missed and found inactive routes, the demand on AODV can be represented by merely the loss of path to the gateway instead of waiting for data to come for transmission. Thus, the standard was modified to directly start route discovery after path loss in these cases: the period ALLOWED_HELLO_LOSS × HELLO_INTERVAL was elapsed without receiving any packets; detection of link loss; receiving RERR message; an expired entry is expunged from the routing table.
- The drone broadcasts RERR with a delay equals the average value of the path discovery time to allow for completing the discovery process of a drone in a failed path before its precursors start their discoveries.
- The time before which an invalidated route cannot be deleted from the table is decreased by putting K = 3 in the equation of computing the DELETE_PERIOD.
- Because the main cause of route discovery process failure is the loss of RREP, and instead of sending RREP-ACK for every RREP, the RREP is sent twice separated by NODE_TRAVERSAL_TIME if the discovery failure is repeated two or more times.

**Fig. 22:**
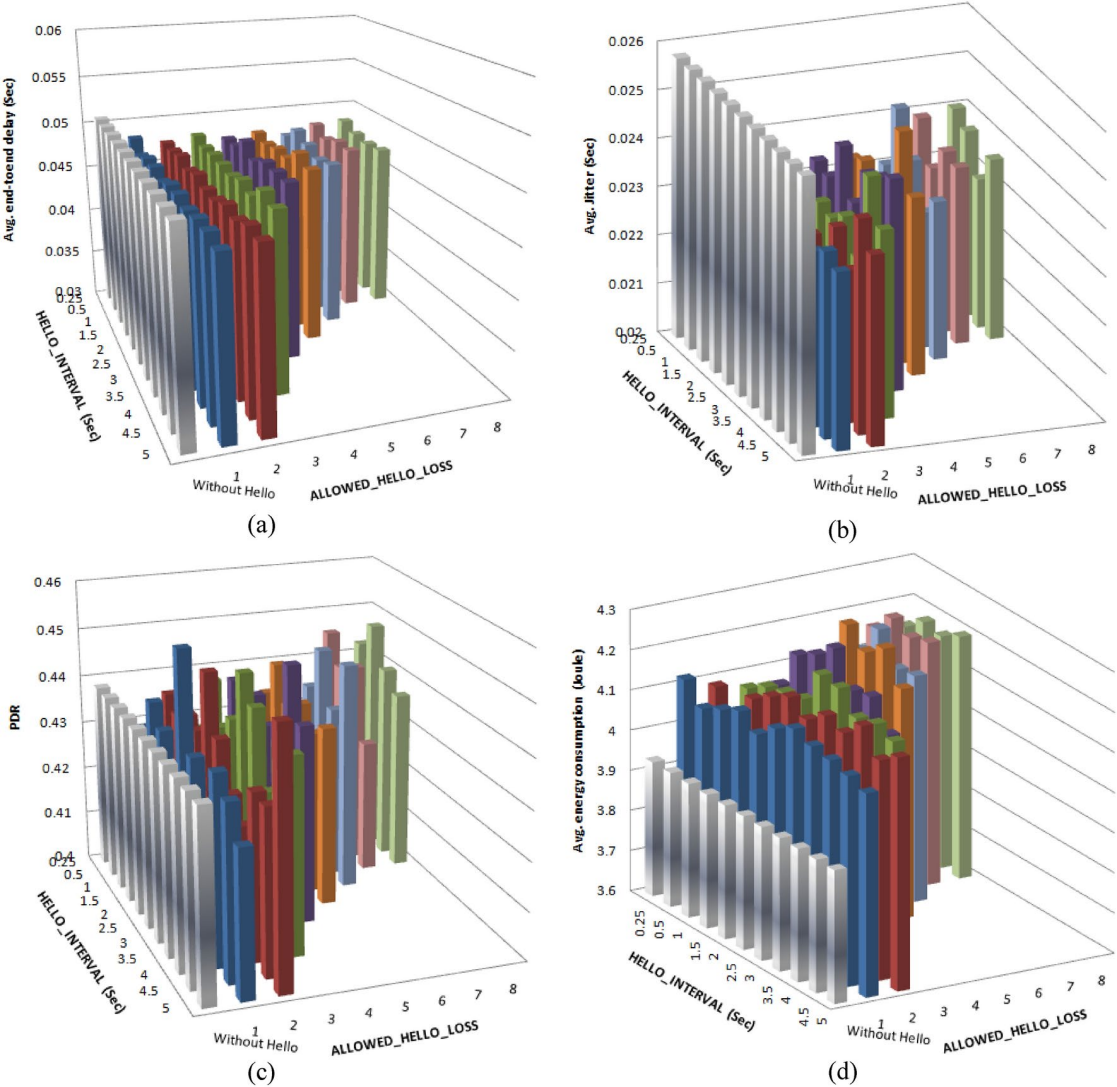
Comparing the performance of the AODV-based FANET, with the proposed modifications and without using Hello, under the recommended operating environment.(a) represents the performance with respect to average end-to-end delay, (c) represents the performance with respect to average jitter, (c) represents the performance with respect to packet delivery ratio, and (d) represents the performance with respect to average energy consumption.

The results obtained after applying the modifications are depicted in Fig. 22. From the obtained results and the analytical decision model, the best solution of the modified protocol, which is corresponding to ALLOWED_HELLO_LOSS = 6 and HELLO_INTERVAL = 1.5 Sec, now causes an average jitter smaller than the first scenario result by about 11.6%, and its average delay is also decreased by about 7.4%; the PDR value wasn’t affected, while the performance encounters an increase in energy consumption by about 5.5%.

### The recommended FAODVN-OE against the default values

First the number of drones is varied around the tested value to find a range of recommended drone density; the corresponding figures were omitted for the sake of paper length. All four parameters show an increasing linear trend with the nodes number. While preserving the jitter limit, it was found that the 20–24 drones number range achieves the required performance, with a delay doesn’t exceed 0.055 Sec, a 8% increase in energy consumption, and a 20.5% increase in PDR. The recommended FAODVN-OE is indicated in Table 3..

**Table 3:** The recommended operating environment of AODV-based FANET.

FAODVN-OE	
**Operating conditions**	**Values of parameters**
Density = 20 drone/km^3^—24 drone/km^3^	ACTIVE_ROUTE_TIMEOUT = 13 Sec
	NET_DIAMETER = 2
	NODE_TRAVERSAL_TIME = 0.01 Sec
	TTL_THRESHOLD < = TTL_START + TTL_INCREMENT
Speed = 5 m/s—10 m/s	TTL_START = 2
	TTL_INCREMENT = 2
	RREQ_RETRIES = 1
Transmission interval = 5 Sec—10 Sec	**Modified HELLO related values of parameters**
	ALLOWED_HELLO_LOSS = 6
	HELLO_INTERVAL = 1.5 Sec
	$$\text{DELETE}\_\text{PERIOD}= 3\times \text{ ACTIVE}\_\text{ROUTE}\_\text{TIMEOUT}$$

**Fig. 23:**
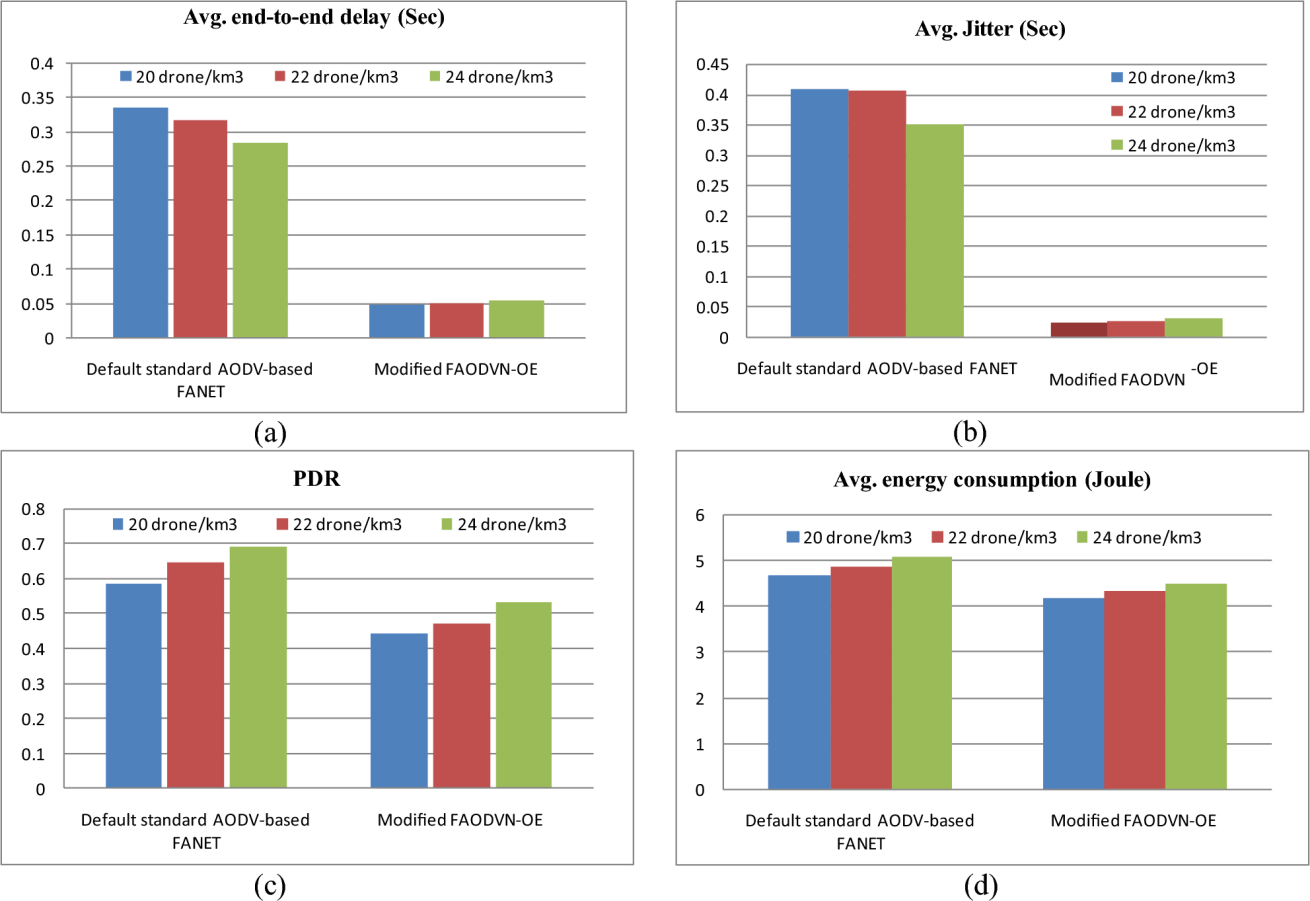
FAODVN-OE with modifications against the default parameters values and Hello. (a) represents the performance with respect to average end-to-end delay, (c) represents the performance with respect to average jitter, (c) represents the performance with respect to packet delivery ratio, and (d) represents the performance with respect to average energy consumption.

Next in this section, taking the FAODVN-OE into account accompanied by the proposed modifications, the performance of the network is tested against its performance under the default values of the parameters and when the standard HELLO mechanism is used, moreover the default standard network is tested under the recommended operating conditions. As indicated by Fig. 23, the recommended operating conditions with default parameters values result in high jitter and delay, while the modified FAODVN-OE performance meets the jitter and delay limits. It is succeeded in reducing the jitter and delay by on average 93.2% and 83.8%, respectively, with 10.7% smaller energy consumption at the expense of PDR reduction estimated at 24.5% on average.

## Conclusions and future work

This paper addresses the application of Ad Hoc standard routing protocols to FANET by adjusting the protocol settings; making, if needed, small amendments to the protocol operations; and specifying the operating condition that is suitable to the protocol operation, instead of designing from scratch non-standardized protocols specific to FANET. The popular protocol AODV was selected for this study. Extensive simulations were conducted first to deeply understand the protocol design and study the performance of AODV-based FANET under different operating conditions including drone density, speed, and transmission interval. It was found that the best condition under which AODV can be applied to FANET is high density, low speed range, and quasi-high transmission rate. Then, a parametric study was conducted and the standard was amended by proposing a bundle of modifications. The conditions that were tested to make a decision on sending the periodic Hello message were modified to help in sending Hello messages only when there is a need and in the appropriate time. The meaning of the demand in AODV was redefined to indicate merely a path loss instead of a need to transmit data through a missing path. Based on that a drone that is included in a precursor list a long a reverse path starts directly route discovery when it receives a RERR but with a delay allows for the previous hop to complete its discovery. Another modification made is related to decreasing the DELETE_PERIOD, accelerating the expunge of routes, and speeding up finding an alternative route. Finally, a modification related to sending the RREP where made such that the RREP is sent twice if the path discovery process failed twice one after another. The modified recommended Operational Environment of AODV-based FANET (FAODVN-OE) has been found to achieve high performance in terms of jitter and delay. FAODVN-OE succeeded in reducing jitter and delay by on average 93.2% and 83.8%, respectively. FAODVN-OE causes also less energy consumption, but the PDR was reduced by on average 24.5%. Further analysis of FAODVN-OE behavior and proposing different protocol amendments based on FAODVN-OE pave the way for improvements in PDR preserving jitter and delay target values at acceptable level of energy consumption, and this is an inevitable direction for future work. Building a testbed to apply and test FAODVN-OE is also a direction of future work.

## Abbreviations

MANET: Mobile Ad-hoc Network
FANET: Flying Ad-hoc Network
AODV: Ad Hoc On-demand Distance Vector
FAODVN-OE: Flying Ad-hoc On-demand Distance Vector Network Operational Environment
GBS: Ground Base Station
GPS: Global Positioning System
CSMA/CA: Carrier-sense Multiple Access with Collision Avoidance
TDMA: Time Division Multiple Access
PDR: Packet Delivery Ratio

## Declarations

### Data availability

"All data generated or analysed during this study are included in this published article".
